# Effect of umbilical cord milking versus delayed cord clamping on preterm neonates in Kenya: A randomized controlled trial

**DOI:** 10.1371/journal.pone.0246109

**Published:** 2021-01-26

**Authors:** Mandeep Sura, Alfred Osoti, Onesmus Gachuno, Rachel Musoke, Frank Kagema, George Gwako, Diana Ondieki, Patrick M. Ndavi, Omondi Ogutu

**Affiliations:** 1 Department of Obstetrics and Gynecology, College of Health Sciences, University of Nairobi, Nairobi, Kenya; 2 Department of Global Health, The University of Washington, Seattle, Washington, United States of America; 3 Department of Pediatrics, College of Health Sciences, University of Nairobi, Nairobi, Kenya; 4 Department of Obstetrics and Gynecology, Kenyatta National Hospital, Nairobi, Kenya; RCSI & UCD Malaysia Campus (formerly Penang Medical College), MALAYSIA

## Abstract

**Background:**

Delayed cord clamping (DCC) is a placental to new-born transfusion strategy recommended by obstetric and gynaecological societies. Though not widely adopted, umbilical cord milking (UCM) may achieve faster transfusion when DCC cannot be performed such as when a neonate requires resuscitation.

**Methods:**

Pragmatic, two-arm, randomized clinical trial in which consenting women in spontaneous labour or provider-initiated delivery at 28 to less than 37 weeks at Kenyatta National Hospital in Nairobi, Kenya, were enrolled. At delivery, stable preterm infants were randomized to UCM (4 times) or DCC (60 seconds). Neonatal samples were collected for analysis at 24 hours after delivery. Maternal primary PPH (within 24 hours) and neonatal jaundice (within 1 week) were evaluated clinically. The primary outcome was the mean neonatal haemoglobin level at 24 hours after birth. Modified Intention to treat analysis was used for all outcomes. P-value was significant at p<0.05.

**Results:**

Between March 2018 to March 2019, 344 pregnant women underwent screening, and 280 eligible participants were randomized when delivery was imminent. The intervention was not performed on 19 ineligible neonates. Of the remaining 260 neonates, 133 underwent UCM while 128 underwent DCC. Maternal and neonatal baseline characteristics were similar. The mean neonatal haemoglobin (17.1 vs 17.5 grams per decilitre, p = 0.191), haematocrit (49.6% vs 50.3%, p = 0.362), anaemia (9.8% vs 11.7%, p = 0.627), maternal PPH (2.3% vs 3.1%, p = 0.719) were similar between UCM and DCC respectfully. However, neonatal polycythaemia (2.3% vs 8.6%, p = 0.024) and neonatal jaundice (6.8% vs 15.6%, p = 0.024) were statistically significantly lower in UCM compared to DCC.

**Conclusion:**

UCM compared to DCC for preterm neonates resulted in similar outcomes for neonatal haemoglobin, haematocrit, anaemia and maternal primary PPH and a lower proportion of neonatal polycythaemia and clinical jaundice. UCM offers a comparable method of placental transfusion compared to DCC and may be considered as an alternative to DCC in preterm neonates at 28 to <37 weeks’ gestation.

## Introduction

A global call to action, to end preventable deaths of newborns by 2030 is embodied within the sustainable development goals [1]. Preterm birth (PTB) and its complications are an important cause of neonatal deaths (1.1 million annually) and the second leading cause of death for under-5’s [2]. Worldwide, the PTB rate is estimated at 10.6% with approximately 78% occurring in sub-Saharan Africa and Southeast Asia [3]. The national prevalence of PTB in Kenya and locally at Kenyatta National Hospital is 12.3% and 18.3% respectively [4].

A prevalent quandary seen in preterm neonates is anaemia of prematurity. Several studies are now focusing on the effect of placental to newborn transfusion strategies in an effort to achieve maximum haemoglobin at birth, which has been associated with reduced mortality and short-term complications. Two strategies have been explored, viz. Delayed cord clamping (DCC) and intact umbilical cord milking (UCM). The American College of Obstetricians and Gynaecologists recommends a 30–60 second delay in umbilical cord clamping for all preterm deliveries as an effective method of reducing anaemia of prematurity [5]. For preterm infants, benefits include the better establishment of red blood cell volume and decreased need for blood transfusions, as well as lowering the incidence of intraventricular haemorrhage (IVH) and necrotizing enterocolitis [6]. DCC has received much attention in recent years, however, the one-minute delay has limited use in neonates with impaired hemodynamic transition requiring resuscitation, hence the need for more exploration into intact UCM. Intact UCM improves pulmonary blood flow immediately at birth, assisting with lung expansion at the onset of respiration therefore facilitating earlier onset of breathing compared to DCC [7]. Theoretical risks exist such as; over transfusion, symptomatic polycythaemia and jaundice. UCM has been shown to have an increased risk of severe IVH in the extreme preterm infants [8]. This has created some reluctance in the clinical application of this method of placental to newborn transfusion.

Though not widely adopted, intact UCM may achieve faster transfusion when DCC cannot be performed such as when a neonate requires resuscitation. We sought to compare preterm (< 37 weeks) neonatal haemoglobin, haematocrit, polycythaemia, anaemia, clinical jaundice and maternal primary post-partum haemorrhage (PPH) following umbilical cord milking versus delayed cord clamping.

## Methods

### Study design and oversight

This was a parallel, pragmatic, randomised controlled trial of intact umbilical cord milking (intervention) versus delayed cord clamping (control) in a ratio of 1:1. The trial protocol was developed by the authors and approved by the Kenyatta National Hospital/University of Nairobi Ethical Review Committee (KNH-UON-ERC), P677/11/2017. Ethical principles expressed in the Declaration of Helsinki were used to conduct the trial. Verbal and written informed consents were sought from each participant. Adequate explanation and counselling were done before attaining consent. Due to the nature of the study, Antenatal clinic (ANC) consent was not practical due to the unpredictability of PTB and generally erratic ANC attendance; majority of the consent was taken while the mother was already in labour. Although this is a vulnerable period for the mother, the nature of such a condition did not allow for earlier consent. The consent process was thorough and delicately handled so as not to overwhelm the mother. This process was free from coercion and any patient who declined consent was excluded from the study. Study participants were monitored by study assistants and clinicians in the respective wards. An independent data safety monitoring board (DSMB) was formed, whose members reviewed trial safety and progress. Safety and progress reports were submitted to the KNH-UON-ERC in case of occurrences of any adverse events.

### Study setting

This study was carried out at the Kenyatta National Hospital (KNH) labour ward, maternity theatres, post-natal wards, new born unit (NBU) and the haematology laboratory. The KNH is the largest public referral and teaching hospital in Kenya receiving patients from Nairobi and its environs as well as referrals from all other hospitals in Kenya. It has an average of 1,000 deliveries per month. These deliveries occur amongst mothers of varying socioeconomic status. The standard of care after delivery for newborns in KNH is DCC and even though UCM is not contraindicated it is rarely practiced. Neonates fitting to the admission criteria of birth weight < 1900 and other medical conditions were managed in the NBU. There was availability of a neonatal ICU (NICU) at KNH in case a neonate required advanced care. The NICU is equipped with a limited number of ventilation machines. Surfactant is available although majority of the patients are unable to afford the cost.

### Study population

Entry criteria included mother-baby pairs between 28 to < 37 weeks gestational age. Gestational age was confirmed by use of last menstrual period dating or if available a first-trimester ultrasound scan. Further to validate the gestational age, a Ballard score was undertaken for each newborn in the study. We excluded pregnancies with multiple gestations, neonates with congenital abnormalities, red cell isoimmunisation, HIV and VDRL positive women, advanced stage of labour (>7cm dilated), antepartum haemorrhage including placenta praevia or placenta abruption. Pregnancies in which neonates required resuscitation as a priori during the antenatal and immediate postnatal period as well as patients who were unwilling to undergo randomization were also excluded.

### Randomization

Randomization occurred in blocks to obtain 140 participants in each arm. Block randomization was used in equal blocks of 14 until the total sample size was achieved. For allocation concealment, the randomization instructions were given to the investigator and research assistants in sequentially numbered, opaque, sealed, identical envelopes with an unpredictable allocation code. Randomization was done by the study midwives when delivery was imminent, for mothers in labour or, at the induction of anesthesia for caesarean deliveries.

### Study interventions

Potential study participants were recruited by the attending midwife at the triage area in the labour ward and through the antenatal wards. The subjects were identified and chosen for the study if they met the eligibility criteria. Women were enrolled at the onset of spontaneous labour (cut off point >7 cm cervical dilation) if they were in labour, or when the decision had been made for caesarean delivery for those not in labour.

All consenting participants were monitored as labour progressed until delivery was imminent, when at this point they were immediately randomized into either intervention group or control group.

Once delivery was imminent, the recruited midwife immediately opened a sealed, numbered, opaque envelope containing the treatment allocation. Neonates underwent either intact UCM or DCC. For neonates that underwent intact UCM, with one hand, the cord was pinched and held closest to the placental end, the other hand was used to milk blood towards the infant where at the umbilical end point the cord was held. This served as one milking motion. The cord was then released at the placental end and allowed to refill over 1 to 2 seconds between each milking motion. This was repeated a total of 4 times. After milking, the cord was clamped and cut, and the neonate handed over to the neonatal receiving team.

For neonates that underwent DCC, the cord was clamped and cut after waiting for 60 seconds. The time was recorded using a stop watch timer in the labour ward delivery room for those who had spontaneous vaginal delivery and a stop watch timer in the maternity theatre for those who had a caesarean section. During the intervention the new-born was positioned on the maternal abdomen after vaginal delivery and on the maternal anterior thigh after cesarean delivery.

The participants, research assistants collecting data and the laboratory staff performing analysis of blood samples were blinded but, the study staff were not due to the nature of the intervention.

After the intervention, routine new-born care was initiated. Immediate cord clamping was implemented in the event that neonatal resuscitation was required.

After delivery, the estimated maternal postpartum blood loss was recorded by the midwife by visual estimation, where a quantitative or semi quantitative estimate was made within the immediate post-partum period. This is the method routinely used at KNH to estimate PPH. If the mother was stable, she was then discharged to the post-natal ward. During post-natal care, mothers were interviewed within 24 hours of delivery to complete the questionnaire.

Clinicians and midwives involved in the delivery were trained on the process of intact UCM via clinical tutorials and videos.

With assistance from the paediatrician, who was part of the study team, clinical examination of the neonate was carried out observing for yellow discolouration in the periphery of the ocular conjunctiva and in the oral mucous membranes. Mothers were trained by the paediatrician before discharge on how to detect jaundice and were given the contact information of the principal investigator. This was guided by pictures and videos on how to detect jaundice [9]. Neonatal follow up was until 1 week after delivery, Mothers who were discharged earlier received telephone calls on day three as well as day seven postpartum for neonatal follow-up and data collection.

Neonatal blood samples were collected using a 23-gauge needle from a peripheral vein amounting to 0.5mls. Blood was collected in EDTA tubes (BD Vacutainer®) 24 hours after delivery. The blood samples were transported in a blood transport box by the research assistant to the Kenyatta National Hospital laboratory where they were analysed for a complete blood count using the Sysmex XN-550 automated haematology analyser. The analyser equipment was calibrated with standard calibrators. The internal quality control was set daily every morning, done by running a known quality control sample along with tests, to confirm the validity of the values of the tests. There was no time lag between the withdrawal of blood samples and analysis.

### Outcome measures

The primary outcome was the mean neonatal venous haemoglobin measured at 24 hours of life. The secondary outcomes were the mean neonatal haematocrit at 24 hours of life, the prevalence of neonatal anaemia and neonatal polycythemia at 24 hours of life, the prevalence of neonatal clinical jaundice within the first week of life and the incidence of maternal primary postpartum haemorrhage.

Neonatal anaemia was defined as hemoglobin concentration <2SD below the normal value for gestational age [10]. Neonatal polycythemia was defined as hematocrit concentration >2 SD above the normal value for gestational age [10]. Primary PPH was defined as within 24 hours a blood loss of >500 ml after vaginal or >1000 ml after cesarean delivery.

### Data collection and management

Data were collected using a closed ended pre-tested data extraction form checked for completeness and correctness and entered into Microsoft Access with in-built consistency and validation checks.

Obstetric and medical information were collected from maternal health care records from the time of admission to discharge.

### Statistical analysis

The sample size was calculated on the basis of a previous trial done by Katheria et al, and using the formula by Chow S (Shao J, Wang H. 2008) for a 2 tailed α value of 0.05 and a power of 80%, 128 participants were required in each group to detect a mean difference in haemoglobin of 0.7g/dl with a standard deviation of 2. To account for attrition, an additional 10 percent were added to the total sample size. Therefore, 280 participants (140 in each arm) were required for the study.

The Biostatistician participated in study design and performed all the analysis and was blinded to allocation groups. Modified intention to treat analysis were used where, some patients who were deemed ineligible after randomization or certain patients who never received the intervention were excluded from analysis. Data were locked until completion of the study thereafter exported to STATA version 13.0 for analysis.

Continuous variables were compared using two-sample t-tests and categorical variables compared using chi-squared tests or Fischer’s exact tests. Outcomes were compared between the two arms using t-test for continuous variables and chi-square test of independence for categorical variables. Risk estimates and corresponding 95% confidence intervals were obtained for primary and secondary outcomes. P< 0.05 was considered statistically significant. All statistical assumptions were met, and validation of final analysis was done.

### Trial registration

This trial was registered at the Pan African Trial Registry (PACTR201906770028735). Although the trial was registered on 3^rd^ March 2018, before enrollment of any patient, the approval was obtained later, therefore, the trial has been registered as a retrospective trial. http://www.pactr.org.

## Results

**Fig 1:**
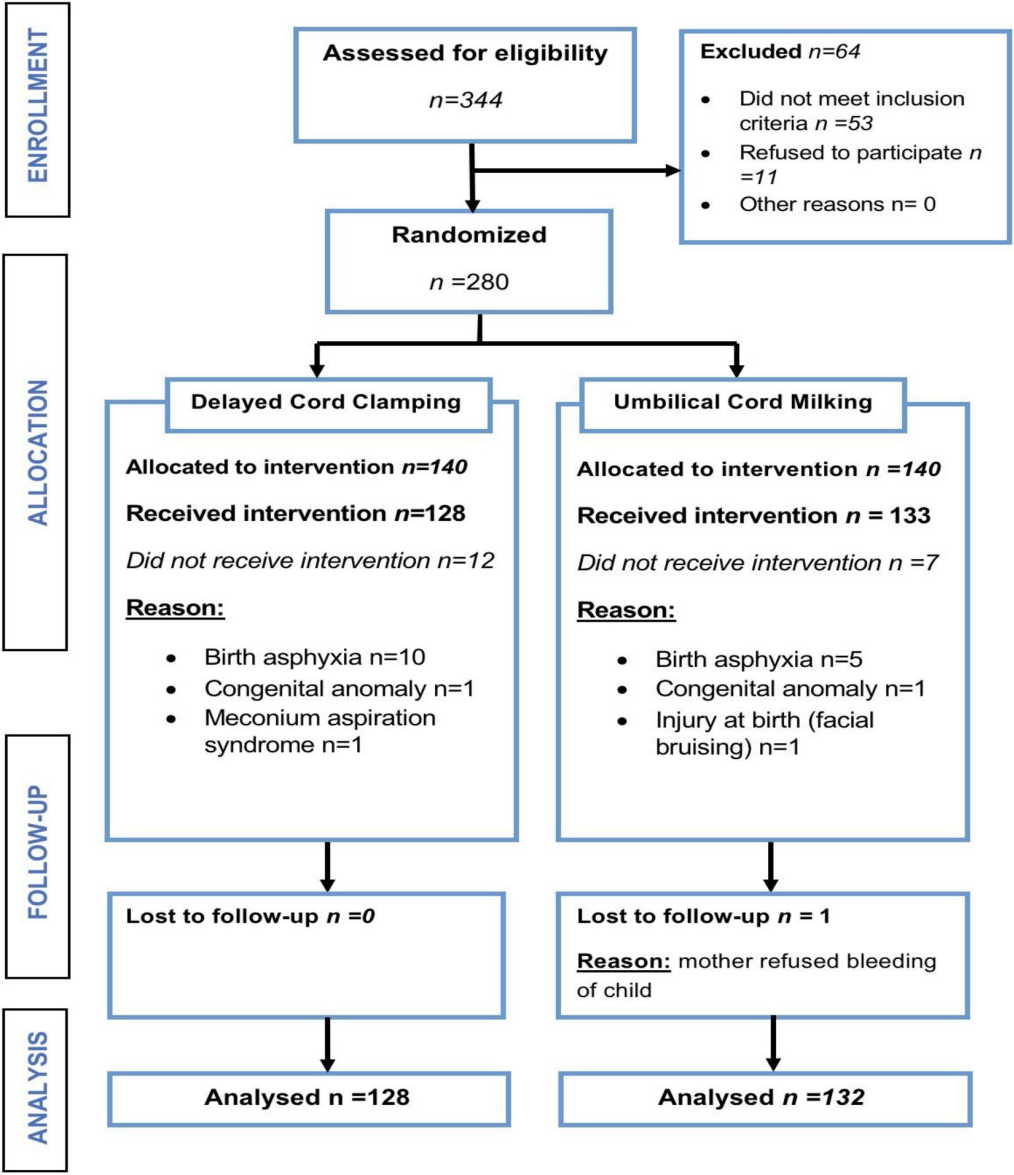
Study enrolment and follow-up of study participants who underwent umbilical cord milking versus delayed cord clamping between March 2018 to May 2019 at Kenyatta National Hospital.

A total of 344 women with suspected preterm labour were screened for the study during the data collection period, and 280 participants were eligible for the study and underwent randomization. Of the 280 mother-baby pairs, 260 neonates completed the trial, 128 in the DCC arm and 132 in the UCM arm (Fig 1 below).

The maternal and neonatal clinical characteristics were comparable between the two study arms therefore randomization was effective (Table 1).

**Table 1:** Baseline sociodemographic and clinical characteristics of participants who underwent umbilical cord milking versus delayed cord clamping between March 2018 to May 2019 at Kenyatta national hospital.

	DCC (n = 140)	UCM (n = 140)
**Age (years)**		
**Mean maternal age**	27.7 ±5.9	27.2 ±5.8
	**n. (%)**	**n. (%)**
18–25	56 (40.0)	69 (49.3)
>25	84 (60.0)	71 (50.7)
**Gravida**		
Primigravida	46 (32.9)	54 (38.6)
**ANC attendance**		
Yes	138 (98.6)	140 (100.0)
**Iron supplementation during pregnancy**		
Yes	107 (76.4)	110 (78.6)
**Mode of delivery**		
Caesarean	49(35.0)	59(41.2)
**Neonatal birth weight**		
**Mean neonatal birth weight (grams) ±SD**	2013.6 ±579.3	2028.8 ±526.2
ELBW (<1000)	5 (3.6)	4 (2.9)
VLBW (1000–1500)	30 (21.4)	26 (18.6)
LBW (1501–2500)	76 (54.3)	87 (62.1)
>2500	29 (20.7)	23 (16.4)
Gender**		
Male	65 (46.4)	71 (50.7)
**Gestational age**		
28 to <32 weeks	39 (27.9)	32 (22.9)
32 to <37 weeks	101 (72.1)	108 (77.1)

There were no statistical differences between the groups for any of the variables.

**One neonate had ambiguous genitalia.

°Plus-minus values are means ±SD. DCC = delayed cord clamping. UCM = intact umbilical cord milking. KNH = Kenyatta national hospital. ANC = antenatal clinic. ELBW = extremely low birth weight. VLBW = very low birth weight. LBW = low birth weight.

The mean haemoglobin as represented in Table 2 showed a statistically non-significant higher (17.5g/dl) value in the DCC arm compared to the UCM arm (17.1g/dl) with a difference of 0.4 g/dl (p = 0.19). The mean haematocrit was higher (50.3%) in the DCC arm compared to the UCM arm (49.6%) with a difference of 0.7% (p = 0.362).

**Table 2:** Clinical and laboratory outcomes of trial participants who underwent umbilical cord milking versus delayed cord clamping between March 2018 to May 2019 at Kenyatta National Hospital.

	UCM *(n = 132)*	DCC *(n = 128)*	Mean difference (95%CI)	p-value
**Mean neonatal haemoglobin g/dl**	17.1 ± 2.2	17.5 ± 2.4	0.4 (-0.2–0.9)	0.191
**Mean neonatal haematocrit g/dl**	49.6 ± 6.4	50.3 ± 6.5	0.7 (-0.8–2.3)	0.362
			**Relative risk (95%CI)**	
**Neonatal polycythaemia^†^ (24 hours of birth)**	3(2.3)	11 (8.6)	0.3 (0.1–0.9)	0.024
**Clinical jaundice (1 week of life)**	9 (6.8)	20 (15.6)	0.4 (0.2–0.9)	0.024
**Neonatal anaemia^‡^ (24 hours of birth)**	13(9.8)	15 (11.7)	0.8 (0.4–1.7)	0.627
**Primary PPH**	3 (2.3)	4 (3.1)	0.7 (0.2–3.2)	0.719

UCM = umbilical cord milking. DCC = delayed cord clamping.

^†^Neonatal polycythaemia: haematocrit levels >2SD above normal value for gestational age.

^‡^Neonatal anaemia: haemoglobin concentration >2SD below the normal value for gestational age. PPH = post-partum haemorrhage.

^§^Primary post-partum haemorrhage: Blood loss of 500 ml or more after spontaneous vertex delivery or 1000ml or more after caesarean section.

Of the neonates in the DCC arm, 8.6% were polycythemic compared to 2.3% in the UCM arm. Infants subjected to UCM had a 70% reduced risk of polycythaemia (RR 0.3; 95% CI: 0.1–0.9; p val. = 0.024) (Table 2).

Neonatal jaundice was higher (15.6%) in the DCC arm compared to the UCM arm (6.8%) with a RR 0.4 (95% CI: 0.2–0.9; p = 0.024), thus infants subjected to UCM had a 60% risk reduction of neonatal jaundice (Table 2).

The DCC arm had 11.7% of neonates with neonatal anaemia while the UCM arm had 9.8% of neonates with anaemia. Although DCC had a slightly higher prevalence of anaemia, the difference was not statistically significant p = 0.627 (Table 2).

The DCC arm had 3.1% of mothers with primary PPH while the UCM arm had 2.3% of mothers with primary PPH. No Statistical significance was noted (p = 0.719) therefore, both arms were equal in incidence of primary post-partum haemorrhage (Table 2).

## Discussion

In this randomized trial involving preterm neonates born at the Kenyatta National Hospital in Kenya, we demonstrated that umbilical cord milking compared to delayed cord clamping resulted in similar outcomes for neonatal haemoglobin, haematocrit, anaemia and maternal primary postpartum haemorrhage. Polycythaemia and clinical jaundice were higher in neonates in the delayed cord clamping arm.

While our findings for mean haemoglobin and haematocrit levels were similar to those reported by Rabe et al and Shirk et al, another study by Katheria et al reported a statistically significant higher (16.3g/dl) mean haemoglobin for UCM versus 15.6g/dl in DCC [11–13]; these comparisons were made in neonates at less than 32 weeks gestational age delivered via caesarean section only; whereas our study compared preterm neonates at less than 37 weeks gestational age delivered both via caesarean section or spontaneous vertex delivery; these differences in gestational age and modes of delivery may account for the contrast in study findings.

Our research found that DCC was associated with an increased prevalence of neonatal polycythaemia in 24 hours differing to findings by Shirk et al [13]. Their study used haematocrit levels above 65% to define polycythaemia, we used gestational age specific reference ranges which might have increased our threshold for detection of polycythaemia [10].

Neonatal jaundice has been highlighted as a risk of placental transfusion. Our findings showed an increased prevalence of clinical jaundice in DCC, contrary to Jaiswal et al that found no significant difference in this prevalence [14]. In our study, the premise for the 60 second delay was to ensure blood transfusion occurs, and while not directly measured, the higher incidence of polycythaemia in the first 24 hours of life may suggest an over-transfusion. Our study was limited in that we did not have actual serum bilirubin measurement, this is not always available at KNH due to intermittent supply of specimen containers and laboratory reagents and transcutaneous estimation is not available. There was also a financial restriction on our part as the financial budget could not cover this cost. For neonates admitted to the NBU, jaundice is mostly a clinical diagnosis on physical examination with a blood test not being routine. Although it was unlikely to miss pathological jaundice, we may have overestimated physiological jaundice.

The prevalence of neonatal anaemia was not statistically significant, similar to study findings by Shirk et al [13]. We found no increased risk in the incidence of maternal primary post-partum haemorrhage. This is consistent with previous systematic reviews that have reported no association between placental transfusion strategies and maternal risk of postpartum haemorrhage [15].

To date, studies are lacking in regards to a suitable position of the neonate during UCM. During DCC, the assumption has been that gravity can facilitate placental transfusion therefore neonates have been held at or below the level of the placenta [16]. A recent trial on term neonates reported that after vaginal delivery, neonates did not have a lower level of transfusion when placed on the maternal abdomen compared to at the level of the introitus [17]. One study reported that preterm neonates held at 15cm above the introitus managed to receive placental transfusion of at least 60% compared to infants held dependent for the same time period and reverse flow did not seem to occur from baby to placenta when holding the neonate at a higher level [18]. Our study placed neonates on the maternal abdomen after vaginal delivery and on the anterior maternal thigh after cesarean delivery, this was in keeping with hospital protocol as well as enhancing maternal-infant bonding. Interestingly, our study shows a higher level of polycythemia in the DCC group suggesting having neonates at a higher level than the placenta had little impact. More studies are needed for determining the optimum position for preterm neonates receiving placental transfusion.

This is the first single center study comparing intact UCM versus DCC in Kenya and to the best of our knowledge within sub-Saharan Africa. This will hopefully encourage further studies, as a feasibility study within the region and similar resource settings has not been done before. In maternities with high patient load, limited theater space, an intervention that can decrease operating time would be useful to explore further. Iron deficiency anaemia represents a major health problem in LMIC country infants therefore an intervention such as UCM to prevent neonatal anaemia would be useful to explore. Our study was limited in that we were unable to follow up neonates for a longer period of time but achieving higher initial haemoglobin levels at birth has shown to be one of the preventive measures of anaemia of prematurity [19].

Current studies are reporting a significantly increased risk of intraventricular hemorrhage (IVH) in very preterm neonates (<28 weeks) undergoing UCM [8]. This population was not included in our study, most of the premies we studied had a mean gestational age of 32 weeks where the risk has been reported to be lower [20]. A recent systematic review reports the risk of severe IVH in preterm infants <34 weeks gestational age [21]. Data on IVH are still limited in gestational groups greater than 28 weeks to confidently exclude any risk and unfortunately due to limitations in availability of cranial ultrasound in our setting, this study was unable to provide any further data on this outcome.

Our study would have also liked to explore other outcomes associated with prematurity such as necrotizing enterocolitis, retinopathy of prematurity and mortality rates in a longer follow-up period. Our follow up period was very short and during this period we did not have any mortality of the neonates receiving either intervention.

## Conclusion

Umbilical cord milking compared to delayed cord clamping resulted in similar outcomes for neonatal haemoglobin, haematocrit, anaemia and maternal primary postpartum haemorrhage. In addition, umbilical cord clamping resulted in a lower proportion of neonatal polycythaemia at 24 hours and neonatal jaundice at one week of life. However, this study was unable to provide any data about severe adverse effects such as IVH. Until further information is known about the potential adverse outcomes, the use of UCM might be an option in preterm infants above 28 weeks’ who need immediate resuscitation. Further studies, particularly in similar resource settings are needed, and these should look specifically for IVH and include long-term outcomes.

## Supporting information

S1 File Consort checklist. (DOC)

S2 File Study protocol. (DOCX)

S1 Data (DOCX)
