# Supplementary material for: Effect of umbilical cord milking versus delayed cord clamping on preterm neonates in Kenya: A randomized controlled trial
Source: PLoS One. 2021 Jan 26;16(1):e0246109. doi: 10.1371/journal.pone.0246109 (PMC7837492; doi:10.1371/journal.pone.0246109)
Supplement: S2 File — (DOCX) [file pone.0246109.s002.docx]

**EFFECT OF UMBILICAL CORD MILKING VERSUS DELAYED CORD CLAMPING ON SELECTED HEMATOLOGICAL AND CLINICAL PARAMETERS IN PRETERM NEONATES AT KENYATTA NATIONAL HOSPITAL: A RANDOMIZED CONTROLLED TRIAL.**

**Dr. MANDEEP SURA**

**H58/80772/2015**

**Department of Obstetrics and Gynecology,**

**The University of Nairobi.**

**Proposal submitted in partial fulfillment for the award of degree in masters of medicine in Obstetrics and Gynecology at the University of Nairobi.**

# CERTIFICATE OF SUPERVISORS

**DR.ONESMUS .W. GACHUNO**, MBchB, M.MED (Obs/Gyn).

SENIOR LECTURER, DEPARTMENT OF OBSTETRICS AND GYNAECOLOGY

CONSULTANT OBSTETRICIAN GYNAECOLOGIST,

UNIVERSITY OF NAIROBI/KENYATTA NATIONAL HOSPITAL.

Signature…………………………………….Date………………………………….

**DR.ALFRED OSOTI,** MBchB, M.MED (Obs/Gyn), MPH

LECTURER, DEPARTMENT OF OBSTETRICS AND GYNAECOLOGY,

CONSULTANT OBSTETRICIAN AND GYNAECOLOGIST,

UNIVERSITY OF NAIROBI/KENYATTA NATIONAL HOSPITAL.

Signature……………………………………….Date……………………………..

**DR.KAGEMA FRANCIS,** MBchB, M.MED (Obs/Gyn)

CONSULTANT OBSTETRICIAN AND GYNAECOLOGIST,

KENYATTA NATIONAL HOSPITAL.

Signature……………………………………….Date……………………………..

**PROF.MUSOKE RACHEL N** M.MED (Pediatrics); Diploma in Neonatology,

SENIOR LECTURER, CONSULTANT PAEDIATRICIAN NEONATOLOGIST,

UNIVERSITY OF NAIROBI/KENYATTA NATIONAL HOSPITAL.

Signature……………………………………….Date…………………………………

# DECLARATION

I do declare that this research is to be undertaken in part fulfillment of the Masters of Medicine in Obstetrics and Gynecology from the University of Nairobi and will be my original work and has not been undertaken and presented for a degree in any other university.

Signed:……………………………………….… Date :……………………………………………………

**Dr. Mandeep Sura**

**Department of Obstetrics and Gynaecology**

**University of Nairobi.**

Contents

[CERTIFICATE OF SUPERVISORS ii](#_Toc499215451)

[DECLARATION iii](#_Toc499215452)

[LIST OF ABBREVIATIONS vi](#_Toc499215453)

[OPERATIONAL DEFINITIONS OF TERMS vii](#_Toc499215454)

[LIST OF TABLES AND FIGURES viii](#_Toc499215455)

[ABSTRACT ix](#_Toc499215456)

[Introduction/background 1](#_Toc499215457)

[LITERATURE REVIEW 2](#_Toc499215458)

[Introduction and epidemiology 2](#_Toc499215459)

[Anaemia of prematurity 2](#_Toc499215460)

[Haemoglobin levels at birth and outcomes 3](#_Toc499215461)

[Placental transfusion 3](#_Toc499215462)

[Umbilical cord milking 3](#_Toc499215463)

[Delayed cord clamping 4](#_Toc499215464)

[Risks and benefits 5](#_Toc499215465)

[CONCEPTUAL FRAMEWORK 6](#_Toc499215466)

[Conceptual framework narrative 7](#_Toc499215467)

[Justification 8](#_Toc499215468)

[Study objectives 9](#_Toc499215469)

[RESEARCH METHODOLOGY 10](#_Toc499215470)

[STUDY DESIGN 10](#_Toc499215471)

[STUDY LOCATION: 10](#_Toc499215472)

[STUDY PERIOD 10](#_Toc499215473)

[STUDY POPULATION: 10](#_Toc499215474)

[SAMPLE SIZE: 12](#_Toc499215475)

[PATIENT RECRUITMENT PROCEDURE 13](#_Toc499215476)

[Consent 13](#_Toc499215477)

[Randomization 13](#_Toc499215478)

[Blinding 14](#_Toc499215479)

[Intervention groups: 14](#_Toc499215480)

[Enrolment 16](file:///C:\Users\Sura\Desktop\proposal\13th%20draft.docx#_Toc499215481)

[Analysis 16](file:///C:\Users\Sura\Desktop\proposal\13th%20draft.docx#_Toc499215482)

[Allocation 16](file:///C:\Users\Sura\Desktop\proposal\13th%20draft.docx#_Toc499215483)

[DATA COLLECTION 17](#_Toc499215484)

[Maternal data 17](#_Toc499215485)

[Neonatal data 17](#_Toc499215486)

[Blood samples 17](#_Toc499215487)

[Clinical jaundice 17](#_Toc499215488)

[DATA VARIABLES 18](#_Toc499215489)

[DATA COLLECTION AND MANAGEMENT 19](#_Toc499215490)

[DATA ANALYSIS 19](#_Toc499215491)

[ETHICAL CONSIDERATIONS 20](#_Toc499215492)

[Ethical Review 20](#_Toc499215493)

[Informed consent 20](#_Toc499215494)

[Risks 21](#_Toc499215495)

[Benefits 21](#_Toc499215496)

[Confidentiality 21](#_Toc499215497)

[Study discontinuation 21](#_Toc499215498)

[Training 22](#_Toc499215499)

[STUDY LIMITATIONS 23](#_Toc499215500)

[STUDY TIMELINE 24](#_Toc499215501)

[BUDGET 25](#_Toc499215502)

[REFERENCES 26](#_Toc499215503)

[Dummy tables 30](#_Toc499215504)

[APPENDICES 33](#_Toc499215505)

[APPENDIX 1: CONSENT 33](#_Toc499215506)

[APPENDIX II: INCLUSION AND EXCLUSION SCREENING ENROLMENT FORM 37](#_Toc499215507)

[APPENDIX III: DATA COLLECTION TOOL 38](#_Toc499215508)

[APPENDIX IV: DATA AND MONITORING SAFETY PLAN 42](#_Toc499215509)

[APPENDIX V: CALL LOGS OF FOLLOWUP INTERVIEWS: 45](#_Toc499215510)

# LIST OF ABBREVIATIONS

AMTSL= Active management of third stage of labour

ACOG= American college of obstetricians and gynaecologists

ATP= Adenosine tri phosphate

DCC= Delayed cord clamping

EDTA= Ethylenediaminetetraacetic acid

ERC= Ethical review committee

EPO= Erythropoietin

HB= Haemoglobin

HCT= Haematocrit

ICC = Immediate cord clamping

IVH= Intraventricular haemorrhage

KDHS= Kenya Demographic and health survey

KNH= Kenyatta National Hospital

MCV= Mean corpuscular volume

MCHC= Mean corpuscular haemoglobin concentration

MCV= Mean corpuscular volume

NEC= Necrotizing enterocolitis

PCV= Packed cell volume

PPH = Post partum haemorrhage

RBC= Red blood cell

RCT= Randomized controlled trial

UON= University of Nairobi

WHO= World health organization

# OPERATIONAL DEFINITIONS OF TERMS

- **Anaemia:** Hb concentration more than two standard deviations (SD) below the mean of similarly aged infants from an iron-supplemented reference population
- **Anaemia in pregnancy:** defined as Hb < 11 g/dl Ref sources
- **Clinical jaundice:** yellowish discolouration of the skin, and/or conjunctiva.
- **Delayed cord clamping (Late cord clamping)**: clamping the umbilical cord between 60-120 seconds after birth. Ref sources
- **Early cord clamping/ Immediate cord clamping:** Clamping of the umbilical cord within 30 seconds of birth.
- **Neonatal Haematocrit:** Range of normal between 45-65%
- **Placental transfusion**: The transfer of residual placental blood to the baby during the first few minutes of age.
- **Polycythaemia:** Haematocrit levels >2SD above the normal value for gestational age.
- **Preterm infant:** infant born less than 37 weeks’ gestation
- **Primary post-partum haemorrhage:** blood loss of 500mls after vaginal delivery or 1000mls after cesarean delivery; within in the first 24 hours after delivery.
- **Umbilical cord milking:** the unclamped umbilical cord is grasped and blood is pushed toward the infant several times before it is clamped to auto infuse blood into the preterm neonate

#

# LIST OF TABLES AND FIGURES

[Figure 1: Conceptual Framework 6](#_Toc499198607)

[Figure 2: CONSORT diagram: study enrolment, intended randomization. 16](file:///C:\Users\Sura\Downloads\13th%20draft.docx#_Toc499198608)

# ABSTRACT

**Background:** The placenta is a useful reservoir of fetal blood that can serve to alleviate anaemia of prematurity, prevalent in almost all premature neonates(1). Immediate cord clamping is a routine practice worldwide despite current evidence on the benefits of providing placental transfusion to the new born(2). Umbilical cord milking and delayed cord clamping are two such strategies providing for placental transfusion. Major obstetric and gynaecological societies are now recommending delayed cord clamping(3,4). Umbilical cord milking is a cost effective intervention and can be considered for faster placental transfer when delayed cord clamping cannot be performed such as when a neonate requires resuscitation. The findings of this study will inform strategies to reduce neonatal anaemia in a country that is one of 15 responsible for over 2/3^rd^ the world’s preterm births and its subsequent complications specifically anaemia(5).

This study aims to evaluate that umbilical cord milking is comparable in benefits to delayed cord clamping and can be beneficial to premature neonates where every second after birth is essential.

**Objective:**  To determine the effects of umbilical cord milking versus delayed umbilical cord clamping on selected haematological and clinical parameters in preterm neonates.

**Methodology:** Randomized controlled trial, single blind. Mother-baby pairs at premature gestation (<37weeks) who meet the selection criteria, will be randomized to either Umbilical cord milking (n=140) or Delayed cord clamping (n=140). UCC will be performed≥60 seconds after delivery and UCM will be performed 4times.

The primary outcomes of the study will be haemoglobin and haematocrit levels determined 24 hours after birth. The secondary outcomes will be neonatal jaundice and maternal primary postpartum haemorrhage.

**Study setting:** Kenyatta National hospital labour ward, maternity theatre and neonatal intensive care unit.

**Data collection tool**: Interviewer administered questionnaire will be used by the principal investigator and 2-research assistants.

**Analysis plan**: Levels of haemoglobin and haematocrit of newborn infants following umbilical cord milking will be compared to those of newborns who undergo delayed cord clamping using unpaired students T- test and a P value <0.05 for significance. Prevalence will be compared using proportions and chi squared test of independence. Measurements of excess risk will be estimated using relative risk at 95%confidence interval that excludes the null value will be considered significant. Intention to treat analysis will be used.

# Introduction/background

Over the decades, immediate cord clamping has become routine practice in majority of delivery facilities. This practice was an adjunct to Active management of third stage of labour in an initiative package to reduce post partum haemorrhage and was adopted worldwide. Immediate cord clamping is no longer considered a beneficial component of Active management of third stage of labour and may even be harmful to mothers and babies(6). Immediate cord clamping may deprive the neonate of placental respiration and transfusion resulting in complete asphyxia pending the lungs becoming functional, and 30-50% of the neonate’s natural blood volume. A neonate that receives full placental transfusion will have enough iron stores while immediate clamping may lead to anaemia during the first year of life(7). Several studies are now focusing on the effect of placental transfusion to the new born using two strategies which are Delayed cord clamping and Umbilical cord milking.

The American college of obstetricians and gynaecologists recommend a 30-60 second delay in umbilical cord clamping for all preterm deliveries(8). It is an extremely effective method of enhancing arterial oxygen content, increasing cardiac output and improving oxygen delivery. For preterm infants benefits include better establishment of red blood cell volume and decreased need for blood transfusion as well as lowering incidence of intraventricular haemorrhage and necrotizing enterocolitis(8).

A Preterm infant has impaired hemodynamic transition and needs an alternate method to provide placental transfusion while allowing for resuscitation(9). Umbilical cord milking is appropriate when delayed cord clamping cannot be performed to effect placental transfer.

A prevalent quandary seen in a high percentage of preterm neonates is anaemia which has major adverse health, social and economic development consequences. The placenta is a useful reservoir of fetal blood that could prove useful to the neonate. It is estimated that globally 273 million children under 5 years of age were anaemic in 2011, and about 42% of these cases were attributable to iron deficiency anaemia(10). Children are particularly vulnerable to iron deficiency anaemia because of their increased iron requirements during periods of rapid growth, especially in the first 2 years of life.

Children with iron deficiency are more likely to have delayed psychomotor development, and when they commence school, they are more likely to have impaired psycho motor performance equivalent to a 5–10-point deficit in intelligence quotient(4). Both epidemiological and experimental data suggest that when these impairments occur at an early age, they may be irreversible, even after repletion of iron stores, thus reinforcing the importance of interventions that may increase placental transfer to the neonate.

# LITERATURE REVIEW

### Introduction and epidemiology

Ensuring healthy lives and promoting well being for all and at all ages is part of the sustainable development goals adopted by the international community in the year 2015(11). One of the targets is to end preventable deaths of newborns and children under 5 years of age by 2030 with all countries aiming to reduce neonatal mortality to as low as 12 per 1000 live births and under 5’s to as low as 25 per 1000 live births(11). Pre term birth is the single most important cause of neonatal deaths and second leading cause of death under 5’s(12).

The global Preterm birth rate is estimated at 11.1%(5). Out of 15 million pre term births each year 1.1 million die from preterm birth complications. More than 60 percent of worldwide preterm births occur in sub Saharan Africa and south East Asia(5). The estimated national rate of preterm birth for Kenya is 12.3% that is approximately 193,000 children born prematurely and Kenya is amongst 15 countries that account for two third of the world’s pre term births(5,13).

In a 2011 study, Wagura found the pre term birth rate to be 18.3% at Kenyatta national hospital(14). Statistics show approximately 648 neonates die annually due to prematurity related complications at Kenyatta National Hospital.

### Anaemia of prematurity

Pre term infants are at risk of developing short term and long term complications that result from functional and anatomic prematurity during the neonatal period. One such complication is anaemia of prematurity(1).

During embryogenesis, fetal erythrocytes are produced in the yolk sac for the first few weeks and by the end of the first trimester the fetal liver becomes the primary site of erythropoiesis. By approximately 32 weeks gestational age the liver and the bone marrow are responsible for erythropoiesis until finally by week 40 of gestation the bone marrow is solely responsible. Erythropoietin being the most important erythropoietic growth factor is initially produced in the fetal liver and as gestation advances production shifts to the kidneys. Stimulation is in response to anaemia and tissue hypoxia. The fetal liver has a higher threshold for stimulation compared to the fetal kidneys consequently production of erythropoietin is blunted in an anaemic premature whose primary site is the fetal liver. Additionally Iron and other substrates are required to mount a response to erythropoietin which may be insufficient in premature neonates(15).

The average life span of a neonatal RBC is approximately 60 days(16) ranging from one half to two third that of an adult RBC (120 days). This is due to reduced levels of intracellular ATP, enzyme activities and carnitine. The RBC has increased susceptibility to fragmentation of the red cell membrane and increased susceptibility to lipid peroxidation(17).

### Haemoglobin levels at birth and outcomes

Factors such as low haematocrit, mean blood pressure, urine output and high oxygen requirement during the first 24 hours of life have been shown to influence short term outcomes for infants(18). Research by Banerjee et al has shown that low haemoglobin at birth is an independent risk factor for mortality and probability of receiving blood transfusion in preterm infants at<32 weeks gestation. Improving infants blood volume and haemoglobin level at birth may reduce mortality and short term complications of prematurity(19).

One of the goals of improving essential neonatal care is to deliver adequate oxygen to meet tissue demand. Increasing fetal haemoglobin by placental transfusion is an extremely effective method of enhancing arterial oxygen content, increasing cardiac output and improving oxygen delivery(20). Oxygen delivery is dependent upon cardiac output, haemoglobin concentration oxygen carrying capacity of haemoglobin and arterial oxygen saturation. Anaemic preterm infants may be less able to maintain oxygen delivery.

### Placental transfusion

Placental transfusion is the transfer of residual placental blood to the baby during the first few minutes of age. The goal of placental transfusion is to facilitate transfer of blood volume from placenta to the newborn.

The fetus has an approximate blood volume of 70ml/kg and the placenta a further 45ml for each kg of fetal weight. If the umbilical cord is not clamped immediately after birth a volume of 20-35ml/kg can be transfused to the fetus potentially representing 50% increase in fetal blood volume(21).Two placental transfusion techniques are umbilical cord milking and delayed cord clamping.

### Umbilical cord milking

A recent systematic review and meta-analysis of 7 randomized clinical trials of umbilical cord milking in infants delivered at <33 weeks demonstrated that infants that undergo umbilical cord milking have higher haemoglobin and lower risk of oxygen requirement and intraventricular haemorrhage of all grades compared with immediate cord clamping(22). Another meta-analysis by Dang D et al showed that by facilitating the early stabilization of blood pressure, UCM at preterm birth was found to be comparatively safe and associated with lower blood transfusion exposure and lower incidence of IVH, NEC and death(23).

A study done by kateria et al showed that umbilical cord milking produced greater placental transfusion, demonstrated by higher initial haemoglobin, higher blood pressure and improved systolic blood flow and urine output for infants delivered by caesarean delivery(24).

Cord milking before clamping improves pulmonary blood flow immediately at birth and assists lung expansion at the onset of respirations. Milking assisted with earlier onset of breathing compared with delayed cord clamping(20). Cord milking may offer an advantage over delayed cord clamping in neonates who are considered too unstable to wait for delayed cord clamping and are at higher risk for intraventricular haemorrhage or death.

Umbilical cord milking can be performed in any low resource setting and provides adequate placental transfusion without delay making it feasible for infants requiring immediate resuscitation. It is an easy intervention with potential to improve neonatal outcome. Milking the cord increases the initial haematocrit and may lessen the need for transfusion in the neonatal period(25).

One published randomized clinical trial has compared umbilical cord milking with immediate cord clamping in extremely preterm deliveries(less than 28 weeks) showed that umbilical cord milking reduced the need for red blood cell transfusions in the neonatal period, increased infants blood pressure in the first 12 hours of life and urine output in the first 72 hours of life(26).

### Delayed cord clamping

The American college of obstetrician and gynaecologists recommend delay cord clamping in healthy infants for 30-60seconds. Benefits in the preterm infants include improved transitional circulation, better establishment of red cell volume, decreased need for blood transfusion and lower incidence of necrotizing enterocolitis and intraventricular haemorrhage(27,28). WHO recommends umbilical cord not to be clamped earlier than one minute after birth in term and pre-term infants(29). The American academy of paediatricians recommends delayed cord clamping for at least 30-60 seconds for term and preterm infants(30).

In a large clinical trial that compared delayed cord clamping with neonatal venous haematocrit levels and clinical outcome at term, it was found that prevalence of haematocrit(<45%)anaemia was lower in groups assigned cord clamping at 1 minute and 3 minutes compared to 15 seconds. The intervention seemed to reduce the rate of neonatal anaemia(31).

Delayed cord clamping allows for expansion of blood volume from autologous placental blood to provide 60% more red blood cells and 30% more blood volume than immediate cord clamping. For preterm neonates this translates to better cardiopulmonary adaptation to extra uterine life and diminished need for red cell transfusions(32).

### Risks and benefits

The greatest barriers to clinical application of placental transfusion are theoretical risks such as over transfusion, symptomatic polycythemia, jaundice, hypothermia and delayed resuscitation. None of these risks have appeared in the current randomized controlled trials and meta-analysis on term or preterm infants (20,33,34).

Too many red blood cells has been a concern but red cells and blood volume expand together, increasing capillary beds throughout the body(35).

Recent meta analysis shows no difference in clinical jaundice(34). There was a slight increase in the need for phototherapy in delayed cord clamping(2%) but recent evidence that higher levels of bilirubin within normal limits may offer neuroprotection(36).

In a study by Rabe H et al, a prospective cohort study, showed milking of the cord 4 times did not have any long-term adverse effect on neurodevelopmental outcome, suggesting that cord milking could be used as an alternative to delayed cord clamping(37)

Many maternity care providers continue to clamp the umbilical cord immediately after an uncomplicated vaginal birth, even though the significant benefits of DCC are now well known. In some cases this continued practice is due to a misunderstanding of placental physiology in the first few minutes after birth. In others, human nature plays a role: we are often reluctant to change the way we are taught to do things, even in the face of clear evidence. Though there is no scientific support for ICC, entrenched medical habits can be glacially slow in changing. Often heard objections are that immediate cord clamping helps prevent post partum haemorrhage but there is no convincing evidence to support this.

Studies have shown no increase in the incidence of postpartum haemorrhage from delayed umbilical cord clamping(34). Concerns regarding maternal risks become particularly relevant in high risk pregnancy in which the benefits of DCC need to be balanced with the timely resuscitation of the woman such as in cases of haemorrhage from placenta previa or placental abruption after delivery of an infant.

# CONCEPTUAL FRAMEWORK

Premature Neonates

(28-36weeks and 6 days)

PREDISPOSED TO ANAEMIA

1. Inadequate red blood cell production.
2. Shortened red blood cell life span.
3. Iron deletion impairing recovery from anaemia.

METHODS TO INCREASE PLACENTAL TRANSFER

UMBILICAL CORD MILKING

DELAYED CORD CLAMPING

- Active placental transfer of cord blood.
- Takes 20 seconds.
- Improves circulatory volume at birth
- Higher systemic blood pressure in neonates improving oxygenation.
- Higher red blood cell levels
- Passive placental transfer of cord blood.
- Between 1-3 minutes
- Improves circulatory volume at birth.
- Increases oxygen carrying capacity to tissues
- Improves iron statues up to 6months.

- Increased Haemoglobin
- Increased Haematocrit
- Decreased Neonatal Anaemia
- Decreased post partum haemorrhage
- Decreased jaundice

Figure 1: Conceptual Framework

# Conceptual framework narrative

Developmentally determined physiological processes predispose the premature neonate directly to anaemia. These factors are inadequate red blood cell production, shortened red blood cell life span and iron depletion impairing recovery from anaemia.

The placenta is a subsidiary reservoir of fetal blood that could prove useful to the predisposed neonate. Placental transfusion is a useful strategy that can be used to increase placental transfer of vital nutrients to the neonate. Two such strategies that can be employed to increase of placental transfusion are umbilical cord milking and delayed cord clamping.

Both strategies have similarities in the physiological processes that aim to be beneficial to the neonate except that delayed cord clamping is a passive process and takes place over 1-3 minutes whereas umbilical cord milking is through active placental transfer and takes approximately 20 seconds.

The study seeks to compare selected haematological and clinical outcomes from both strategies. Umbilical cord milking may replicate or increase benefits by two fold amongst preterm neonates compared to delay cord clamping.

# Justification

Neonatal mortality is the leading cause of mortality in children under the age of five years in Kenya. Nearly 35% of under-5 deaths occurred during the neonatal period; 1/3^rd^ of all neonatal deaths are due to severe infections, followed by birth asphyxia, preterm births and congenital anomalies.

Preterm neonates are at risk of developing complications that will affect their chances of survival. The extent to which they develop complications strongly depends on the quality of care they receive at birth. In a preterm neonate already predisposed to such disadvantages the extra blood received from the placenta can give a boost to their already disadvantaged status.

The additional blood obtained through placental transfer can improve haemoglobin levels at birth, stabilize neonatal transition, reduce rates of blood transfusion and increase neonatal iron storage in preterm neonates.

Delayed cord clamping is recommended by all major obstetric societies but less information exists on umbilical cord milking.UCM is potentially an effective, faster placental transfer strategy when DCC cannot be performed such as when a neonate requires resuscitation or to expedite delivery in the advent of maternal complications.

Directly comparing the recently recommended practice of delayed cord clamping to umbilical cord milking before clamping has potential to prove beneficial for the neonates in question, as well inform obstetric practice and policy on placental transfusion in a maternity with heavy workloads.

If this study shows that umbilical cord milking has the same benefits if not more to the preterm neonate, this can give clinicians confidence to implement and use umbilical cord milking as part of their management plan for preterm neonates in a maternity with a high turnover of patients. It can also be used to advocate for policies to introduce umbilical cord milking as a routine practice to be done on all preterm neonates. Currently no specific protocol with regards to placental transfusion exists in Kenyatta national hospital.

Most studies have been done in the developed countries; this study seeks to replicate similar or better outcomes for haemoglobin levels amongst a population with higher incidence of anaemia.

# Study objectives

Research question:

Is there a difference in haemoglobin, haematocrit and neonatal jaundice between preterm infants who will undergo umbilical cord milking versus delayed cord clamping?

Broad objective:

To determine the effects of umbilical cord milking versus delayed cord clamping on haemoglobin, haematocrit, and neonatal jaundice of preterm neonates.

Primary objectives:

Among preterm neonates at 28-36 weeks who will undergo umbilical cord milking versus delayed cord clamping at vaginal or caesarean delivery, to:

1. Determine and compare the mean haemoglobin and haematocrit values at 24 hours of life.

Null hypothesis: There is no difference in the mean haemoglobin and haematocrit values 24 hours after birth comparing pre term neonates delivered at 28-36 weeks who will undergo umbilical cord milking versus delayed cord clamping.

1. Determine and compare the incidence rate of neonatal polycythemia at 24 hours of life.

Null hypothesis: There is no difference in the incidence rate of neonatal polycythemia at 24 hours after birth comparing pre term neonates delivered at 28-36 weeks who will undergo umbilical cord milking versus delayed cord clamping.

1. Determine and compare the incidence rate of neonatal jaundice within the first week of life.

Null hypothesis: There is no difference in the incidence rate of neonatal jaundice within the first week of life comparing pre term neonates delivered at 28-36 weeks who will undergo umbilical cord milking versus delayed cord clamping.

Secondary objectives:

Among preterm neonates at 28-36 weeks who will undergo umbilical cord milking versus delayed cord clamping at vaginal or caesarean delivery, to:

1. Determine and compare the incidence rate of neonatal anaemia within the first 24 hours of life.
2. Determine and compare the incidence rate of maternal primary post partum haemorrhage within 24 hours of delivery.

# RESEARCH METHODOLOGY

### STUDY DESIGN

Single blind, open-label, Randomised controlled trial.

This will be a parallel group study with 1:1 randomisation. Application of block randomization will be used with 140 participants in each arm comparing neonatal venous haemoglobin and haematocrit values between umbilical cord milking and delayed cord clamping among preterm babies delivered at Kenyatta National Hospital, Nairobi 24 hours after birth.

### STUDY LOCATION:

This study will be carried out at the Kenyatta National Hospital (KNH) labour ward, maternity theatres, post natal wards and the neonatal intensive care unit. The KNH is the largest teaching and referral hospital in East and Central Africa and within its complex are College of Health Sciences (University of Nairobi); the Kenya Medical Training College; Kenya Medical Research Institute and National Laboratory Service (Ministry of Health).

KNH has 50 wards, 22 out-patient clinics, 24 theaters (16 specialized) and Accident & Emergency Department. Out of the total bed capacity of 2,009,196 of the beds are for obstetric services. It is located in the Upperhill area of the capital city of Kenya, Nairobi in Nairobi County and serves the lower and middle class population.

### STUDY PERIOD

This study will be carried out at Kenyatta National hospital from December 2017 to June 2018.

### STUDY POPULATION:

**INCLUSION CRITERIA**:

This will include:

- Mother-baby pairs between 28 to <37 weeks gestational age. Gestational age will be confirmed by use of Last Menstrual Period dating or if available a first trimester ultrasound scan will be used. After delivery a Ballard score will be done by the paediatrician at 24 hours of life to validate the gestational age.
- Preterm deliveries occurring for both elective indications and spontaneously through either vertex delivery or caesarean section.
- Reassuring fetal status.
- Informed consent must be provided.

**EXCLUSION CRITERIA**:

- Pregnant women with multiple gestations.
- Neonates with congenital abnormalities(e.g. neural tube defects)
- Red cell isoimmunisation.
- Seropositive serology for HIV and VDRL.
- Ante partum haemorrhage including Placenta accrete or placenta abruption.
- Need for neonatal resuscitation.
- Tight nuchal cord necessitating early cutting.
- Patients who are incapable of informed consent (severely ill), or unwilling to undergo randomization.

# SAMPLE SIZE:

Using the formula for sample size calculation (Chow S, Shao J, Wang H. 2008).

$$n=\frac{r+1}{r}\frac{{{SD}^{2}\left( Z_{\beta}+Z_{\alpha/2} \right)}^{2}}{d^{2}}$$

Where:

SD **=** standard deviation from a previously published study=2

d=expected mean difference between the groups

r=ratio for the groups= 1:1

$Z_{\beta}$**=**standard normal variation for level of power=80% power is 0.84

Z$\alpha/2$= standard normal variation for level of significance= 1.96

In a similar study done by Katheria AC(24), using the mean value for haemoglobin for UCM and DCC, we get our sample size as follows:

$$n=\frac{1+1}{1}\frac{{2^{2}\left( 0.84+1.96 \right)}^{2}}{{0.7}^{2}}$$

n= 128 per arm

To account for attrition bias an additional 10 percent will be added to the total sample size.

Therefore a total sample size of 280 will be required for the study.

# Study procedures

### PATIENT RECRUITMENT

Potential study participants will be recruited by the attending midwife at the triage area in labour ward as well as though the antenatal wards on a daily basis. The subjects are identified and chosen for the study if they meet the eligible criteria (inclusion and exclusion). Women will be enrolled at onset of spontaneous labour (cut off point <7 cm cervical dilation) if they are in labour or when the decision has been made for caesarean delivery for those not in labour. Recruitment and enrolment will be carried out by the research assistants, attending midwife or principal investigator who are all part of the study team.

### Consent

Once identified, the principal investigator or research assistant will brief the patients on the purpose and method of the study and verbal consent will be attained. Thereafter Consent will be given in written form, on a pre designed consent form. The consent form provided will describe the purpose of the study, the study procedure to be followed as well as the potential benefits and risks of participating in the study. Any pertinent questions regarding the study from the parent/guardian will be answered at this point.

This process will be free from coercion and will be explicitly voluntary.

Those who accept to take part in the study will be asked to sign the consent form which will be counter signed by the investigator. Records will be kept regarding reasons for non participation of eligible participants. The investigator or research assistant will then countersign the consent form. A copy of the signed consent form will be given to the participant.

A log will be available in Labour ward of the principle investigators, research assistants and enrolled patients. Data will be then collected from the eligible patients and every entry signed in the log book.

All consenting patients who meet the eligible criteria will be monitored as labour progresses until delivery when they will be immediately randomized into the two different intervention groups.

### Randomization

Randomization will be done by the midwives carrying out the interventions who are part of the study in labour ward. Randomization will be done when delivery is imminent. This is characterised by urge to bear down or second stage of labour. For caesarean delivery randomization will be done at induction of anaesthesia in the operating theatre.

The statistician will use block randomization in equal blocks of 14 until the total sample size is achieved. They will be randomized into two groups. A random allocation sequence will be done on computer generated slips of paper bearing the allocation code which will be kept in serially numbered opaque sealed envelopes with an unpredictable allocation code. Recruited patients will be randomly assigned to either the control arm or the intervention arm in a 1:1 ratio.

Using STATA statistical package randomization ado package, computer generated random sequences in blocks of 4 and 6 will be used to determine the order of intervention allocation and avoid anticipation of the allocation group for the next patient while maintain a balance of the patients recruited in each of the arms at each point. The randomly generated list of the allocation sequence will be printed on small cards each representing the allocation of each patient in the order generated by the software. Each of these cards will be sealed in opaque envelopes that will be sequentially numbered in the order of the list generated.

After identifying an eligible patient and consent granted the next sequentially numbered envelope will be opened and then will determine which arm the patient will be allocated. Each participant will be assigned a unique 4 digit number for subject identity and confidentiality.

### Blinding

The nature of the intervention makes it impossible to blind the clinicians performing the intervention. Although study staff will not inform the mothers of their allocation, the nature of the intervention makes it impossible to blind them. If an already randomized mother later becomes ineligible, the assigned allocation code will not be re-used. The principle investigator or the research assistants will monitor the delivery and therefore will not be blinded to treatment assignment.

The laboratory staff performing analysis of blood samples will be blinded to the allocation group.

### Intervention groups:

A structured survey questionnaire will be used to gather obstetrical and medical details from mothers that meet study criteria during the first stage of labour. The questionnaire will be filled in by the research assistants or principal investigator. Once delivery is imminent, the midwife will immediately open a sealed, numbered, opaque envelope containing the treatment allocation. Neonates will undergo either umbilical cord milking or delayed cord clamping.

For neonates undergoing UCM, after delivery the cord will be pinched as close to the placenta as possible and milked towards the infant over 2 second duration. The cord is then released and allowed to refill over 1 to 2 seconds between each milking motion. This is repeated a total of 4 times. After milking the cord will be clamped and handed over to the neonatal receiving team.

For neonates undergoing DCC, the cord will be clamped after waiting 60 seconds. Timing will be noted by using a stop watch timer in the labour ward delivery room and a wall mounted clock in the maternity theatre.

The position of the new born during DCC or UCM will be on the maternal abdomen for both caesarean delivery as well as after spontaneous vaginal delivery.

Early care of the new born should be initiated, including drying and stimulating for first breath or cry. Maintain normal temperature with skin to skin contact and covering the infant with dry linen. Secretions should be cleared if only copious or appear to be obstructing the airway.

DCC and UCM should not interfere with active management of third stage of labour including Uterotonics agents after delivery of the newborn to minimize bleeding.

If placental circulation is not intact, such as in the case of placental abruption, abnormal placentation, and cord avulsion as well as Newborns without spontaneous breathing during the first 10 seconds of life, with major congenital malformations diagnosed at birth and with tight nuchal cord will be subjected to early cord clamping based on need for resuscitation, regardless of the assigned intervention.

Clinicians and midwives involved in the delivery will be trained on the process of UCM by live clinical tutorials and videos.

All patients irrespective of which group they are in will be reviewed by consultants and senior house officers during the ward rounds.

### DATA COLLECTION.

### Maternal data

Obstetric and medical information will be collected from maternal health care records from the time of admission to antenatal care these included reported illness, medication, parity, blood group Rhesus factor status, and Haemoglobin values. This data will be collected by the research assistants or principal investigator. After giving birth, the estimated maternal postpartum blood loss will be recorded by the midwife within two hours after delivery. During postnatal care, mothers will be interviewed within 24 hours of delivery in the post natal ward to further to complete the questionnaire.

#### Neonatal data

The research assistants will measure the time from complete delivery of the baby to the first clamp on the umbilical cord with a stopwatch or using the wall clock. The infant will be taken care of as per normal protocol after delivery once allocated intervention has been carried out. Blood samples for a full blood count will be obtained 24 hours after delivery in the neonatal ward by the phlebotomist. Data will be collected by the research assistants and filled in on the data collection form.

#### Blood samples

All neonatal blood samples will be collected using a 23-gauge needle from a peripheral vein, amounting 0.5mls by the attending paediatric resident as per the hospital protocol. Blood will be collected in EDTA tubes (BD Vacutainer®).The blood sample will be transported in a blood transport box by the research assistant to the Kenyatta national hospital laboratory and analysed for ‘complete blood count’: Hb, Hct, MCV, mean cell haemoglobin concentration (MCHC). Complete blood counts will be analysed using the automated haematology analyser. The results will be collected by the research assistant and the data filled into the data collection form by the research assistant.

#### Clinical jaundice

With assistance from the paediatrician who will be part of the study team, clinical examination of the neonate will be carried out observing for yellow discolouration in the periphery of the ocular conjunctiva and in the oral mucous membranes (under the tongue, hard palate). Information will be filled on the data collection form by the research assistant. Mothers will be trained before discharge on how to detect jaundice and contact information will be given to them. Mothers will be trained by the paediatrician on how to detect jaundice by way of pictures and videos on how to detect jaundice(38).Telephone calls will be made to participants on day three as well as day seven postpartum regarding neonatal follow-up for those discharged earlier. Contact information of principal investigator will be given to the mothers if they wish to contact the principal investigator at any time in-between the follow up calls.

### DATA VARIABLES

- Primary dependent variables; the infants’ venous haemoglobin and haematocrit value will be measured at 24 hours of life. Polycythemia, Neonatal jaundice and need for phototherapy/exchange transfusion
- Secondary dependent variables: neonatal anaemia, maternal primary post partum haemorrhage.
- Independent variables: Delayed cord clamping (control) and Umbilical cord milking (intervention).

#


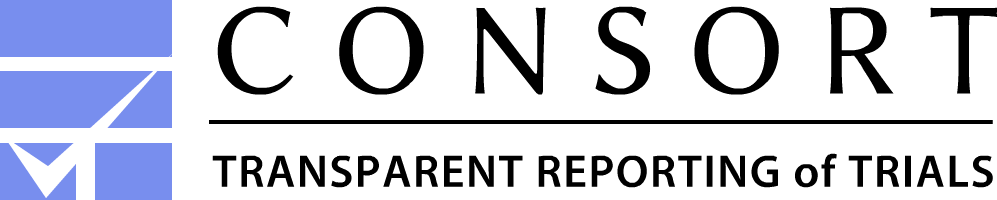


Excluded (n= )

- Multiple gestation (n= )
- Declined consent (n= )
- Major congenital abnormalities (n= )
- Red cell isoimmunisation(n= )
- Positive serology HIV/Hep/VDRL (n= )
- Tight nuchal cord (n= )
- Ante partum haemorrhage (n= )
- Need for neonatal resuscitation (n= )

Assessed for eligibility (n= )

## Enrolment

Figure 2: CONSORT diagram: study enrolment, intended randomization.

## Analysis

Analysed for primary and secondary outcomes (n= )
♦ Excluded from analysis (give reasons) (n= )

Analysed for primary and secondary outcomes (n=)
♦ Excluded from analysis (give reasons) (n= )

Vaginal delivery (n= )

Caesarean delivery (n=)

## Allocation

Randomized delayed cord clamping (n= )

Randomized to

Umbilical cord milking (n= )

Randomized to

Delayed cord clamping (n= )

Randomized to

Umbilical cord milking (n= )

Randomized (n= )

# DATA COLLECTION, MANAGEMENT AND ANALYSIS

After obtaining formal permission and ethical clearance, the study will be conducted at Kenyatta national hospital. The eligible participants will sign an informed consent. Respective data collection will be done in the Reproductive health labour ward, postnatal wards, paediatric wards and the neonatal ICU. Follow up data will be collected via phone call for the patients that have been discharged from hospital early.

Data will then be entered onto a REDCap electronic database with inbuilt consistency and range checks. No patient-identifiable information will be collected on the questionnaires or entered on to the REDCap database. The database will be password protected and only authorised personnel will have access to the data. The questionnaires will be stored in lockable cabinet and only the PI or the persons granted permission by the PI will have access. Automated haematology analyser will measure haemoglobin and haematocrit, by colorimetric principle.

The principal investigator and the recruited study research assistants will carry out the data collection. A structured survey questionnaire will be used to gather obstetrical and medical details and a venous blood sample will be taken from the neonate for laboratory investigations.

Training of research assistants will take place over the duration of one week; initially they will observe the process of obtaining informed consent and filling of the questionnaires. Thereafter they will work under supervision until the principal investigator is satisfied. The principal investigator will constantly review the questionnaires for completion. The intervention will be carried out by the midwife who will be trained by clinical tutorials and video tutorials.

The data monitoring will be continuous and the statistician will alert the principal investigator of any overt differences in the two groups. The study will be halted if there is evidence of significant statistical advantage in whichever group, to avoid denying the corresponding group an advantageous intervention.

LABORATORY PROCEDURES:

COLLECTION AND TRANSPORT:

All neonatal blood samples will be collected using a 23-gauge needle from a peripheral vein amounting to 0.5mls by the attending paediatric resident as per the hospital protocol. Blood will be collected in EDTA tubes (BD Vacutainer®).The blood sample will be transported in a blood transport box by the research assistant to the Kenyatta national hospital laboratory and analysed for ‘complete blood count’: Hb, Hct, MCV, mean cell haemoglobin concentration (MCHC). Complete blood counts will be analysed using the automated haematology analyser. The results will be collected by the research assistant and the data filled into the data collection form by the research assistant.

QUALITY CONTROL

The analyser equipment will be calibrated with standard calibrators. The internal quality control will be set daily every morning, done by running a know quality control sample along with tests, to confirm the validity of the values of the tests.

INFECTION CONTROL

All infection control protocols will be adhered to throughout the study procedure .This will include aseptic technique for all invasive medical procedures, hand hygiene and use of personal protective equipment for all medical procedures.

### DATA ANALYSIS

Descriptive statistics will be reported to describe the study population. Proportions will be reported for categorical data, the mean and standard deviation will be reported for continuous normal data while the median and Inter-quartile range [IQR] will be reported for non-normal data. Proportions in each study arm will be compared for the secondary outcome.

Data will be analysed using STATA analytical package version 13,while presentation will be inform of charts and graphs for descriptive statistics while tables will be used for the presentation of inferential statistics. All statistical tests will be performed at 5% level of significance (p value<0.05) while odds ratios and corresponding 95% confidence intervals will be reported for regression analysis.

# ETHICAL CONSIDERATIONS

### Ethical Review

This protocol and the template informed consent form found in appendix, and any subsequent modifications to this form, will be reviewed and approved by the Kenyatta National Hospital/University of Nairobi Ethics Research Committee prior to initiation of the study, with respect to scientific content and compliance with applicable research and human subjects regulations.

This protocol, the informed consent form, and any other requested documents, as well as any subsequent modifications, will be reviewed and approved by the Ethical Review Committee.

Safety and progress reports will be submitted to the KNH-UoN ERC, after study completion or in case of study termination or occurrences of any adverse events. The reports will include the total participants enrolled in the study, the number of participants that completed the study, all changes in the research activity, and all other problems that were not anticipated that involves risks to human subject or others. Finally, all open DSMB reports will be provided to the KNH-UoN ERC.

### Informed consent

We will obtain a written informed consent from participants or from the parents/guardians of participants who in one way or the other cannot consent for themselves. Adequate explanation and counseling will be done before consenting. Participant’s partner will be informed about the study. If the participant requests the partner’s presence or advice before consenting, they will be granted if the partner is within the Hospital. They will then append their signature as a witness as provided for in the consent form. However the participant’s approval will be considered as tacit approval from the partner, unless otherwise specified.

The informed consent form describes the purpose of the study, the procedures to be carried out, the risks and benefits in accordance with applicable regulations. The consent form will be translated into Swahili for ease of understanding.

Literate participants will append their signatures at the provided space in the consent form. Non-literate participants will document their approval by marking the form using their thumbprint, in the presence of a literate third party witness. Any other local ERC requirements for obtaining informed consent from non-literate persons will be followed. Participants or their parents/ guardians will be provided a copy of their informed consent forms and this fact will be documented in the participant’s record

### Risks

Risks will be anticipated and addressed accordingly. Side effects of delayed cord clamping and umbilical cord milking are rare. In case the neonate requires immediate resuscitation, this will be attended to.

We will ensure the participants privacy and confidentiality is maintained at all times. However it is possible that others will know of the participant’s involvement in the study, although we think there's no stigma related to this, and any potential harm will be alleviated.

### Benefits

If umbilical cord milking is found to be more beneficial for the premature, they will gain by receiving the best obstetric practice especially in scenarios where time is of importance and neonatal resuscitation is required. The findings will benefit the participants in case the positive findings are implemented at a later date. The information learnt from this study may in future help the participants and others.

### Confidentiality

Information collected to be handled with Belmont’s principles of confidentiality (Respect for persons, Beneficence and Justice). Each participant will be allocated a unique study identification number for confidentiality. The coded number will identify all reports, data collected and other administrative forms. All the information on the participants and the study as a whole will be stored and secured at the study site and stored in locked file cabinets only accessible by study staff. All databases will be secured with password-protected access systems. The study information of the participants will not release without the written permission of the participant, except for monitoring by the DMSB, or KNH-UoN-ERC.

### Study discontinuation

The study's goal is to achieve ≥ 95% participant retention. So we will make every reasonable effort to retain any enrolled study participant to completion of the study. Participants are at will to withdraw from the study if they are unwilling or unable to comply with the required study procedures. In order to protect participants’ safety, the Principal investigator may withdraw study participants from the study.

A final evaluation will be completed for the study participants who withdraw from the study before completion. And the reasons for the withdrawal will be recorded in the participant’s records.

Finally the study may be discontinued at any time by the KNH-UoN-ERC.

### Training

The research team involved will undertake Good Clinical Practice (GCP) training and certification. Once the study has been approved, it will be registered with the clinical trial registry and clinicaltrial.gov.

Consolidated standards of reporting trials (CONSORT) will be used to facilitate complete and transparent reporting of the trial.

Training of research assistants will take place over the duration of one week; initially they will observe the process of obtaining informed consent and filling of the questionnaires. Thereafter they will work under supervision until the principal investigator is satisfied. The principal investigator will constantly review the questionnaires for completion. Research assistants and midwives will undergo training prior to commencement of the study via video tutorials and clinical teachings. Each will undertake good clinical practice certification once the study is approved.

# STUDY LIMITATIONS

The study lacks addition of another control arm to undergo immediate cord clamping. Given the recent evidence on benefits of placental transfusion and adverse effects of ICC there was not enough clinical equipoise to have assigned an ICC arm.

The time period of follow-up is focused on short term outcomes due to timeline limitations, it would be more enriching to have long term outcomes. This cohort can therefore be followed up in another study evaluating the long term outcomes.

The study is not primarily designed to assess possible adverse effects of placental transfusion, however previous studies have disproved a correlation between placental transfusion interventions and PPH, neonatal polycythemia and hyperbilirubinaemia.

It will not be possible to measure the amount of blood actually transfused from the placenta into the newborn child.

# DISSEMINATION OF RESEARCH FINDINGS

All participants in the research will be given a report of the findings, and will be encouraged to comment on them.

As well as the above, dissemination of the results will take place by three methods:

- Production of a report that will be sent to the department of obstetrics and gynaecology as well as the department of paediatrics.
- Publishing papers in specialist and general, national and international journals.
- Presentation of papers at both national and international conferences.

# STUDY TIMELINE

|  |  | | |  |  | | |  | |  | |  |  |
| --- | --- | --- | --- | --- | --- | --- | --- | --- | --- | --- | --- | --- | --- |
|  | **2017** | | **2018** | | | | | | | | | |  |
|  | **August-November** | **December** | **January-February** | | | **March-May** | **June** | | **July** | | **August-** | |  |
| Proposal Development |  |  |  | | |  |  | |  | |  | |  |
| Proposal Presentation |  |  |  | | |  |  | |  | |  | |  |
| Ethics Committee Review |  |  |  | | |  |  | |  | |  | |  |
| Data Collection |  |  |  | | |  |  | |  | |  | |  |
| Data Analysis |  |  |  | | |  |  | |  | |  | |  |
| Results Presentation |  |  |  | | |  |  | |  | |  | |  |
| Publication |  |  |  | | |  |  | |  | |  | |  |

# BUDGET

|  | **Item** | **Amount (Kes)** |
| --- | --- | --- |
| Proposal  Development | Printing: questionnaires | 10,000 |
|  | Opaque envelopes | 2,500 |
|  | Proposal copies | 6,000 |
|  | KNH-UoN ERC | 2,000 |
| Data Collection | Training research assistants | 3,000 |
|  | Research Assistants x 2 pax | 90,000 |
|  | Stationary | 2,500 |
|  | Laboratory costs | 180,000 |
|  | Internet | 15,000 |
|  | Airtime | 5,000 |
|  | Transport/Meetings | 5,000 |
| Equipment | Hypodermic needles, cotton swabs, spirit, EDTA bottles, clean gloves, | 50,000 |
|  | External hard drive |  |
|  |  | 10,000 |
| Data Analysis | Statistician | 50,000 |
| Thesis write up | Printing drafts | 5,000 |
|  | Printing thesis | 6,000 |
|  | Contingency | 30,000 |
|  | TOTAL | **472,000** |

# REFERENCES

1. Strauss RG. Anaemia of prematurity: pathophysiology and treatment. Blood Rev [Internet]. NIH Public Access; 2010 Nov [cited 2017 Nov 9];24(6):221–5. Available from: http://www.ncbi.nlm.nih.gov/pubmed/20817366

2. Downey CL, Bewley S. Historical perspectives on umbilical cord clamping and neonatal transition. J R Soc Med [Internet]. Royal Society of Medicine Press; 2012 Aug [cited 2017 Nov 9];105(8):325–9. Available from: http://www.ncbi.nlm.nih.gov/pubmed/22907549

3. Committee Opinion No. 543. Obstet Gynecol [Internet]. 2012 Dec [cited 2017 Jun 1];120(6):1522–6. Available from: http://content.wkhealth.com/linkback/openurl?sid=WKPTLP:landingpage&an=00006250-201212000-00045

4. II WHO I Guideline: Delayed umbilical cord clamping for improved maternal and infant health and nutrition outcomes. [cited 2017 Jul 5]; Available from: http://apps.who.int/iris/bitstream/10665/148793/1/9789241508209_eng.pdf

5. Born Too Soon Global Map | March of Dimes [Internet]. [cited 2017 Aug 28]. Available from: http://www.marchofdimes.org/mission/global-preterm.aspx#tabs-3

6. Westhoff G, Cotter AM, Tolosa JE. Prophylactic oxytocin for the third stage of labour to prevent postpartum haemorrhage. In: Westhoff G, editor. Cochrane Database of Systematic Reviews [Internet]. Chichester, UK: John Wiley & Sons, Ltd; 2013 [cited 2017 Sep 4]. p. CD001808. Available from: http://www.ncbi.nlm.nih.gov/pubmed/24173606

7. Linderkamp O. Placental transfusion: determinants and effects. Clin Perinatol [Internet]. 1982 Oct [cited 2017 Sep 4];9(3):559–92. Available from: http://www.ncbi.nlm.nih.gov/pubmed/6761038

8. ACOG Recommends Delayed Umbilical Cord Clamping for All Healthy Infants - ACOG [Internet]. [cited 2017 Jun 9]. Available from: http://www.acog.org/About-ACOG/News-Room/News-Releases/2016/Delayed-Umbilical-Cord-Clamping-for-All-Healthy-Infants

9. Katheria AC, Brown MK, Rich W, Arnell K. Providing a Placental Transfusion in Newborns Who Need Resuscitation. Front Pediatr [Internet]. 2017 Jan 25 [cited 2017 Nov 9];5. Available from: http://journal.frontiersin.org/article/10.3389/fped.2017.00001/full

10. Stevens GA, Finucane MM, De-Regil LM, Paciorek CJ, Flaxman SR, Branca F, et al. Global, regional, and national trends in haemoglobin concentration and prevalence of total and severe anaemia in children and pregnant and non-pregnant women for 1995?2011: a systematic analysis of population-representative data. Lancet Glob Heal [Internet]. 2013 Jul [cited 2017 Jul 5];1(1):e16–25. Available from: http://www.ncbi.nlm.nih.gov/pubmed/25103581

11. SDGs .:. Sustainable Development Knowledge Platform [Internet]. [cited 2017 Aug 28]. Available from: https://sustainabledevelopment.un.org/sdgs

12. WHO | Newborn death and illness. WHO [Internet]. World Health Organization; 2011 [cited 2017 Nov 9]; Available from: http://www.who.int/pmnch/media/press_materials/fs/fs_newborndealth_illness/en/

13. PROFILE OF PRETERM AND LOW BIRTH WEIGHT PREVENTION AND CARE. [cited 2017 Aug 28]; Available from: http://www.healthynewbornnetwork.org/hnn-content/uploads/Kenya-1.pdf

14. Health C. PREVALENCE AND FACTORS ASSOCIATED WITH PRETERM BIRTH AT KENYATTA NATIONAL HOSPITAL PETER MWANGI WAGURA MBCh.B. (Moi) H58/68429/2011 RESEARCH DISSERTATION SUBMITTED IN PARTIAL FULFILLMENT FOR THE AWARD OF THE DEGREE OF MASTERS OF MEDICINE IN PAEDIATRICS AND. 2014 [cited 2017 Aug 28]; Available from: http://erepository.uonbi.ac.ke:8080/xmlui/bitstream/handle/11295/74707/Wagura_Prevalence and factors associated with preterm birth at Kenyatta national hospital.pdf?sequence=5&isAllowed=y

15. Ohlsson A, Aher SM. Early erythropoietin for preventing red blood cell transfusion in preterm and/or low birth weight infants. In: Ohlsson A, editor. Cochrane Database of Systematic Reviews [Internet]. Chichester, UK: John Wiley & Sons, Ltd; 2006 [cited 2017 Aug 30]. Available from: http://doi.wiley.com/10.1002/14651858.CD004863.pub2

16. Steiner LA, Gallagher PG. Erythrocyte disorders in the perinatal period. Semin Perinatol [Internet]. NIH Public Access; 2007 Aug [cited 2017 Nov 9];31(4):254–61. Available from: http://www.ncbi.nlm.nih.gov/pubmed/17825683

17. Widness JA. Pathophysiology of Anemia During the Neonatal Period, Including Anemia of Prematurity. Neoreviews [Internet]. NIH Public Access; 2008 Nov 1 [cited 2017 Aug 30];9(11):e520. Available from: http://www.ncbi.nlm.nih.gov/pubmed/20463861

18. Richardson DK, Gray JE, McCormick MC, Workman K, Goldmann DA. Score for Neonatal Acute Physiology: A Physiologic Severity Index for Neonatal Intensive Care. Pediatrics [Internet]. 1993 [cited 2017 Aug 30];91(3). Available from: http://pediatrics.aappublications.org/content/pediatrics/91/3/617.full.pdf

19. Banerjee J, Asamoah FK, Singhvi D, Kwan AWG, Morris JK, Aladangady N. Haemoglobin level at birth is associated with short term outcomes and mortality in preterm infants. BMC Med [Internet]. BioMed Central; 2015 Jan 27 [cited 2017 Aug 25];13:16. Available from: http://www.ncbi.nlm.nih.gov/pubmed/25622597

20. Katheria AC, Lakshminrusimha S, Rabe H, McAdams R, Mercer JS. Placental transfusion: a review. J Perinatol [Internet]. Nature Publishing Group; 2017 Feb [cited 2017 Aug 30];37(2):105–11. Available from: http://www.ncbi.nlm.nih.gov/pubmed/27654493

21. Gutierrez A, Velasquez R, Iriarte M. Analysis of clinical course in term patients with early and delayed umbilical cord clamping after birth. ISPUBCOM Internet J Pediatr Neonatol [Internet]. [cited 2017 Aug 30];11(2). Available from: https://print.ispub.com/api/0/ispub-article/13641

22. Al-Wassia H, Shah PS, T M, E M, AW W, NN F. Efficacy and Safety of Umbilical Cord Milking at Birth. JAMA Pediatr [Internet]. BMJ Publishing, London, England; 2015 Jan 1 [cited 2017 Jul 31];169(1):18. Available from: http://archpedi.jamanetwork.com/article.aspx?doi=10.1001/jamapediatrics.2014.1906

23. Dang D, Zhang C, Shi S, Mu X, Lv X, Wu H. Umbilical cord milking reduces need for red cell transfusions and improves neonatal adaptation in preterm infants: Meta-analysis. J Obstet Gynaecol Res [Internet]. 2015 Jun [cited 2017 Sep 1];41(6):890–5. Available from: http://www.ncbi.nlm.nih.gov/pubmed/25656528

24. Katheria AC, Truong G, Cousins L, Oshiro B, Finer NN. Umbilical Cord Milking Versus Delayed Cord Clamping in Preterm Infants. Pediatrics [Internet]. American Academy of Pediatrics; 2015 Jul [cited 2017 Jun 9];136(1):61–9. Available from: http://www.ncbi.nlm.nih.gov/pubmed/26122803

25. March MI, Hacker MR, Parson AW, Modest AM, de Veciana M. The effects of umbilical cord milking in extremely preterm infants: a randomized controlled trial. J Perinatol [Internet]. NIH Public Access; 2013 Oct [cited 2017 Jun 1];33(10):763–7. Available from: http://www.ncbi.nlm.nih.gov/pubmed/23867960

26. Hosono S, Mugishima H, Fujita H, Hosono A, Minato M, Okada T, et al. Umbilical cord milking reduces the need for red cell transfusions and improves neonatal adaptation in infants born at less than 29 weeks’ gestation: a randomised controlled trial. Arch Dis Child - Fetal Neonatal Ed [Internet]. 2008 Jan 1 [cited 2017 Sep 1];93(1):F14–9. Available from: http://www.ncbi.nlm.nih.gov/pubmed/17234653

27. Rabe H, Diaz-Rossello JL, Duley L, Dowswell T. Effect of timing of umbilical cord clamping and other strategies to influence placental transfusion at preterm birth on maternal and infant outcomes. In: Rabe H, editor. Cochrane Database of Systematic Reviews [Internet]. Chichester, UK: John Wiley & Sons, Ltd; 2012 [cited 2017 Jun 7]. Available from: http://doi.wiley.com/10.1002/14651858.CD003248.pub3

28. American College of Obstetricians and Gynecologists. ACOG committee opinion. American College of Obstetricians and Gynecologists;

29. WHO | Optimal timing of cord clamping for the prevention of iron deficiency anaemia in infants. WHO [Internet]. World Health Organization; 2015 [cited 2017 Jun 8]; Available from: http://www.who.int/elena/titles/full_recommendations/cord_clamping/en/

30. Korioth T. AAP backs delayed umbilical cord cutting for term, preterm infants. AAP News [Internet]. 2017 [cited 2017 Jun 8]; Available from: http://www.aappublications.org/news/2016/12/21/UmbilicalCord122116

31. Ceriani Cernadas JM, Carroli G, Pellegrini L, Otaño L, Ferreira M, Ricci C, et al. The Effect of Timing of Cord Clamping on Neonatal Venous Hematocrit Values and Clinical Outcome at Term: A Randomized, Controlled Trial. Pediatrics [Internet]. 2006 [cited 2017 Jun 8];117(4). Available from: http://pediatrics.aappublications.org/content/117/4/e779.short

32. Strauss RG, Mock DM, Johnson KJ, Cress GA, Burmeister LF, Zimmerman MB, et al. A randomized clinical trial comparing immediate versus delayed clamping of the umbilical cord in preterm infants: short-term clinical and laboratory endpoints. Transfusion [Internet]. NIH Public Access; 2008 Apr [cited 2017 Jun 7];48(4):658–65. Available from: http://www.ncbi.nlm.nih.gov/pubmed/18194383

33. Hutton EK, Hassan ES. Late vs Early Clamping of the Umbilical Cord in Full-term Neonates. JAMA [Internet]. 2007 Mar 21 [cited 2017 Jul 31];297(11):1241. Available from: http://www.ncbi.nlm.nih.gov/pubmed/17374818

34. McDonald SJ, Middleton P, Dowswell T, Morris PS. Effect of timing of umbilical cord clamping of term infants on maternal and neonatal outcomes. In: McDonald SJ, editor. Cochrane Database of Systematic Reviews [Internet]. Chichester, UK: John Wiley & Sons, Ltd; 2013 [cited 2017 Jun 1]. Available from: http://doi.wiley.com/10.1002/14651858.CD004074.pub3

35. Pietra GG, D’Amodio MD, Leventhal MM, Oh W, Braudo JL. Electron Microscopy of Cutaneous Capillaries of Newborn Infants: Effects of Placental Transfusion. Pediatrics [Internet]. 1968 [cited 2017 Sep 1];42(4). Available from: http://pediatrics.aappublications.org/content/42/4/678.short

36. Zahir F, Rabbani G, Khan RH, Rizvi SJ, Jamal MS, Abuzenadah AM. The pharmacological features of bilirubin: the question of the century. Cell Mol Biol Lett [Internet]. 2015 Jan 1 [cited 2017 Sep 1];20(3):418–47. Available from: http://www.ncbi.nlm.nih.gov/pubmed/26208389

37. Rabe H, Sawyer A, Amess P, Ayers S, Brighton Perinatal Study Group. Neurodevelopmental Outcomes at 2 and 3.5 Years for Very Preterm Babies Enrolled in a Randomized Trial of Milking the Umbilical Cord versus Delayed Cord Clamping. Neonatology [Internet]. 2015 Dec 10 [cited 2017 Sep 4];109(2):113–9. Available from: http://www.ncbi.nlm.nih.gov/pubmed/26650133

38. Global Health Media Project – Warning Signs in Newbornsfor mothers and caregivers [Internet]. [cited 2018 Jan 24]. Available from: https://globalhealthmedia.org/portfolio-items/warning-signs-in-newborns-for-mothers-and-caregivers/?portfolioID=5623

# Dummy tables

**Table 1: Maternal baseline characteristics**

| Characteristic | Category | n (%) / mean (SD deviation)  DCC | n(%)/ mean (SD deviation)  UCM | P value |
| --- | --- | --- | --- | --- |
| Socio demographics | | |  |  |
| Age | 18-25  26-35  36-50  >50 |  |  |  |
| Obstetric History | | |  |  |
| Parity | 0  1  2  3  4  5 |  |  |  |
| ANC attendance | Yes  No |  |  |  |
| Facility attended | KNH  Other facility |  |  |  |
| Number of visits | 1  2-4  >4 |  |  |  |
| Gestational age at first visit | |  |  |  |
| Hb(g/dl) at 1^st^ visit | |  |  |  |
| Repeat Hb( If available) | |  |  |  |
| Blood group | A+  B+  AB+  O+  A-  B-  AB-  O- |  |  |  |
| VDRL | Positive  Negative  Unknown |  |  |  |
| HIV | Positive  Negative  Unknown |  |  |  |
| Medications | Magnesium-sulphate  Steroids  Antihypertensive  Narcotics |  |  |  |
| Mode of delivery | Spontaneous vertex delivery  Caesarean section  Assisted vaginal delivery |  |  |  |

**Table 2: Infant baseline characteristics**

| Variable |  | DCC | UCM | P value |
| --- | --- | --- | --- | --- |
| Sex  Gestational age  APGAR score  Birth weight  Birth length  Head circumference  Need for neonatal resuscitation  NICU admission | Male  Female  At 1 min  At 5 min |  |  |  |

**Table 3: Association between delayed cord clamping versus umbilical cord milking and maternal/neonatal complications**

|  | | DCC | UCM | RR | P value |
| --- | --- | --- | --- | --- | --- |
| Maternal | Post partum haemorrhage  Need for blood transfusion |  |  |  |  |
| Fetal | Polycythemia |  |  |  |  |
|  | Neonatal polycythemia |  |  |  |  |
|  | Neonatal jaundice |  |  |  |  |
|  | Need for blood transfusion |  |  |  |  |
|  | Need for phototherapy |  |  |  |  |

**Table 4: neonatal haematological outcomes**

|  |  | DCC | UCM | P value |
| --- | --- | --- | --- | --- |
|  |  | Mean(SD) | Mean(SD) |  |
| Neonatal Hb(g/dl) |  |  |  |  |
| Neonatal Hct (%) |  |  |  |  |
| Serum bilirubin(mg/dl) |  |  |  |  |

# APPENDICES

## APPENDIX 1: CONSENT

Enrolment Identification number _________________

Date (date/month/year):

**Study Title:** **THE EFFECT OF UMBILICAL CORD MILKING VERSUS DELAYED CORD CLAMPING ON SELECTED HAEMATOLOGICAL AND CLINICAL PARAMETERS IN PRETERM NEONATES AT KENYATTA NATIONAL HOSPITAL**

**Principal Investigator:**

**Dr. Mandeep Sura (MBChB)**

**Department of Obstetrics and Gynaecology, University of Nairobi.**

**Tel Number: 0722-775150**

Investigator’s Statement:

We are requesting you and your newborn to kindly participate in this research study. The purpose of this consent form is to provide you with the information you will need to help you decide whether to participate in the study. This process is called ‘Informed Consent’. Please read this consent information carefully and ask any questions or seek clarification on any matter concerning the study with which you are uncertain. You are free to ask any questions about the study. The investigator will be available to answer any questions that arise during the study and afterwards.

Introduction:

Transfer of blood from the placenta to the baby improves both the short-term and long-term blood status for the newborn and does not have clinically significant harmful effects. The best duration of delayed cord clamping appears to be more than 60 seconds, unless the cord stops pulsing sooner. In our setup, average cord clamping time is 20 seconds.

Umbilical cord milking is the active transfer of placental blood and can be achieved in approximately 20 seconds.

Benefits:

As a participant you will benefit from the study by receiving close monitoring. You will benefit by receiving health education and advice on neonatal care. You will be able to access the principal investigator at any time during the study period. Your participation in the study may benefit others in future from the information we find in this study.

Risks:

The possible harms of placental transfer of blood are thought to be secondary to over-perfusion leading to hyper-bilirubinemia and polycythemia, these have been found to be theoretical and no statistical difference in the population has been documented between Delayed cord clamping and Umbilical cord milking.

In case a neonate requires resuscitation, immediate cord clamping is considered so as not to delay resuscitative efforts and this will be given priority.

Voluntariness:

The study will be fully voluntary. There will be no financial rewards to you for participating in the study. One is free to participate or withdraw from the study at any point. Refusal to participate will not compromise you or your child’s care in any way.

Confidentiality:

All the information obtained from you will be held in strict confidentiality. Any information that may identify you or your child will not be published or discussed with any unauthorised persons. No specific information regarding you, your child or your family will be released to any person without your written permission. Your research number will be used in place of your names.

Access of health records

You may apply for access to your own records, or may authorise third parties such as lawyers, employers, or insurance companies to do so on your behalf. The Principal Investigator can be contacted if access to health records is required.

Sharing of results

Study staff will protect your personal information closely so no one will be able to connect your responses and any other information that identifies you. Federal or state laws may require us to show information to university or government officials (or sponsors), who are responsible for monitoring the safety of this study. Directly identifying information (e.g. names, addresses) will be safeguarded and maintained under controlled conditions. You will not be identified in any publication from this study.

Intervention

A structured survey questionnaire will be used to gather your obstetrical and medical details.

When delivery is about to occur, the midwife will immediately open a sealed, numbered, opaque envelope containing the treatment allocation. Two interventions for the newborns will be umbilical cord milking or delayed cord clamping (within 1 minutes after birth). After vaginal or caesarean delivery all babies will be placed on the maternal abdomen, dried and wrapped in a warm towel. The infants will remain in this position until the cord is clamped.

Newborns requiring resuscitation will undergo immediate cord clamping regardless of the assigned intervention. A follow up telephone call will be made to you at three days and one week postpartum to follow on any neonatal complications that may have developed.

You will be able to reach the principal investigator at any time in-between the follow up period.

Problems or Questions:

If you ever have any questions about the study or about the use of the results you can contact the principal investigator, Dr. Mandeep Sura by calling 0722-775150. If you have any questions on your rights as a research participant you can contact the Kenyatta National Hospital Ethics and Research Committee (KNH- ESRC) by calling 2726300 Ext. 44355.

Consent Form: Participant’s Statement:

I having received adequate information regarding the study research, risks, benefits hereby AGREE / DISAGREE (Cross out as appropriate) to participate in the study with my child. I understand that our participation is fully voluntary and that I am free to withdraw at any time. I have been given adequate opportunity to ask questions and seek clarification on the study and these have been addressed satisfactorily.

Parent’s name_____Signature: Date

Thumb print witness name and signature.

I declare that I have adequately explained to the above participant, the study procedure, risks and benefits and given him /her time to ask questions and seek clarification regarding the study. I have answered all the questions raised to the best of my ability.

Interviewer’s name and Signature Date

Problems or Questions:

If you ever have any questions about the study or about the use of the results you can contact the principal investigator, Dr. Mandeep Sura by calling 0722-775150. If you have any questions on your rights as a research participant you can contact the Kenyatta National Hospital Ethics and Research Committee (KNH- ESRC) by calling 2726300 Ext. 44355.

## APPENDIX II: INCLUSION AND EXCLUSION SCREENING ENROLMENT FORM

**Study Title:** **THE EFFECT OF UMBILICAL CORD MILKING VERSUS DELAYED CORD CLAMPING ON SELECTED HAEMATOLOGICAL AND CLINICAL PARAMETERS IN PRETERM NEONATES AT KENYATTA NATIONAL HOSPITAL**

Date: (date/month/year):

Code Number of the Mother-Baby pair: ________________________

Subject Initials: _____________

*Inclusion Criteria: Answers MUST be ‘yes’ for both questions*

1. Gestation between 28 to <37 weeks at time of presentation
2. Live Neonate born via spontaneous vertex delivery or caesarean delivery.
3. Informed consent must be provided.

*Exclusion criteria*: *If any answer is ‘Yes’ exclude from enrolment*

1. Multiple gestations.
2. Placental separation before delivery.
3. Tight nuchal cord necessitating early cutting.
4. Need for neonatal resuscitation.
5. Major congenital abnormalities (e.g. neural tube defects).
6. Red cell isoimmunisation.
7. Positive serology for Human immunodeficiency virus Venereal disease research laboratory.
8. Declined consent.

## APPENDIX III: DATA COLLECTION TOOL

**Study Title:** **THE EFFECT OF UMBILICAL CORD MILKING VERSUS DELAYED CORD CLAMPING ON SELECTED HAEMATOLOGICAL AND CLINICAL PARAMETERS IN PRETERM NEONATES AT KENYATTA NATIONAL HOSPITAL**

**BASELINE QUESTIONNAIRE**

**Part I: Socio demographics**

**Indicate all times using the 24 hour clock, and dates in this format date/month/year.**

DATE__________

Code number of mother-infant pair:_________ Randomization arm: ___

Date of Signed Informed Consent: ____/______/_____

Copy given to patient: Yes / No

1. Age of mother (years) ……………………………….
2. Marital Status

Single □ Widowed □

Married □ Separated □

Divorced □

1. Level of Education

Primary □ Secondary □ Tertiary □

1. Employment status

Self employed □ Salaried employment □ Unemployed □

**Part II: Obstetric History**

1. Parity ………………………
2. Obstetric History

|  | Date  (Year) | Place  Home or HF* | GA** at delivery | Mode of  Delivery | Maternal  Complications | Neonatal  Outcome |
| --- | --- | --- | --- | --- | --- | --- |
| 1 |  |  |  |  |  |  |
| 2 |  |  |  |  |  |  |
| 3 |  |  |  |  |  |  |

*HF-Health Facility GA** Gestational age

1. ANC attendance Yes □ No □
2. If yes, facility attended: KNH □ Other facility □
3. Number of visits …………………………..
4. Date of first visit ./….../……..
5. Gestational age at first visit (in completed weeks) ………/40
6. Iron supplementation during pregnancy Yes □ No □
7. Duration of Iron supplementation…………
8. ANC Profile:

Haemoglobin (g/dl) ……………

Blood group: ………….. Rh: ……………

VDRL:

HIV:

**Part Ill: Index Admission**

1. Date of Admission ……/……./…….
2. Time of admission ……………….
3. Referral status

Referred from other facility □

Self referred □

Booked for delivery at KNH □

1. Gestational age at delivery (in completed weeks) ……../40
2. Gestational age calculated by Dates □ Quickening □

Ultrasound □ Fundal Height □

First clinic visit estimate □

1. Maternal Haemoglobin levels at admission ……………..
2. Maternal PCV at admission ………….
3. Maternal use of drugs for medical condition yes no
4. State which illness medication is used for in Q22 above.

**Part IV: Infant characteristics**

1. Gestational age (weeks)………………
2. Time of delivery …………
3. Time from admission to delivery (days)…………….. Or hours (if less than 24 hours)…………..
4. Live birth □ Still birth □
5. Admitted to NBU Yes □ No □
6. If Yes, diagnosis………………………………….
7. Sex of infant: Males □ Female □

1. Apgar score at 5 minutes

< 7 □ >7-10 □

1. Birth weight (g)…………………

1. Head circumference (cm)………………..
2. Neonatal haemoglobin at 24 hours of life:
3. Neonatal haematocrit at 24 hours of life:

**Part V: Follow up**

MATERNAL COMPLICATIONS

Post partum haemorrhage yes □ No □

Post partum anaemia Yes □ No □

Post partum sepsis Yes □ No □

NEONATE COMPLICATIONS

Clinical Jaundice Yes □ No □

Polycythemia Yes □ No □

Neonatal sepsis Yes □ No □

## APPENDIX IV: DATA AND MONITORING SAFETY PLAN

**Data and Safety Monitoring Plan**

Study Title: **THE EFFECT OF UMBILICAL CORD MILKING VERSUS DELAYED CORD CLAMPING ON SELECTED HAEMATOLOGICAL AND CLINICAL PARAMETERS IN PRETERM NEONATES AT KENYATTA NATIONAL HOSPITAL**

Principal Investigator: Dr Mandeep Sura

**MEMBERS**

Prof. Wamalwa- Acting chairman Paediatric department, UON.

Dr. Rose Kosgei- Obstetrician and Epidemiologist.

Dr.Hussein Dossajee- Statistician.

**BRIEF STUDY OVERVIEW**

**Objective**: To determine the effects of umbilical cord milking versus delayed umbilical cord clamping on haemoglobin and haematocrit level of pre term newborns.

**Methodology**: This will be a randomized controlled trial that will include mother-baby pairs at pre term gestation who meet the selection criteria, 140 newborns will be randomized to delayed umbilical cord clamping (≥60 seconds after delivery as the control group) and 140 newborns will be randomized to early umbilical cord milking (as the experiment group) and their haemoglobin and haematocrit level determined.

**DSMB OVERSIGHT RESPONSIBILITIES**

Oversight of the trial is provided by the DSMB.Meetings will take place to monitor on safety of patients and signals of efficacy, futility or harm. The DSMB members will have a first meeting before study is commenced. A meeting will be constituted in case of any adverse event and a final meeting on conclusion of the study. In case of unacceptable safety concerns/results occur, the board can recommend termination of the study. The safety of the participant is paramount.

**MONITORING PROCEDURES**

Dr. Sura will assure that informed consent is obtained prior to performing any research procedures, that all subjects meet eligibility criteria, and that the study is conducted according to the ERC-approved research plan.

Study data are accessible at all times for the PI to review. The PI will review study conduct every alternate day that is acquisition of consent, any dropouts, and completeness of questionnaire. The PI will review AEs individually real-time and in aggregate on a daily basis.

The PI will ensure all protocol deviations, AEs, and SAEs are reported to the ERC and KNH administration according to the applicable regulatory requirements.

**COLLECTION AND REPORTING OF SAEs AND AEs**

For this study, the following standard AE definitions are used:

**Adverse event:** Any unfavourable and unintended sign (including an abnormal laboratory finding), symptom or disease temporally associated with the use of a medical treatment or procedure, regardless of whether it is considered related to the medical treatment or procedure.

**Serious Adverse Event:** Any AE that results in any of the following outcomes:

- Death
- Life-threatening
- Event requiring inpatient hospitalization or prolongation of existing hospitalization
- Persistent or significant disability/incapacity

AEs are graded according to the following scale:

**Mild:** An experience that is transient, & requires no special treatment or intervention. This includes transient laboratory test alterations.

**Moderate:** An experience that is alleviated with simple therapeutic treatments. Includes laboratory test alterations indicating injury, but without long-term risk.

**Severe:** An experience that requires therapeutic intervention. If hospitalization (or prolongation of hospitalization) is required for treatment it becomes an SAE.

The study uses the following AE attribution scale:

**Not related:** The AE is clearly not related to the study procedures (i.e., another cause of the event is most plausible and/or a clinically plausible temporal sequence is inconsistent with the onset of the event).

**Possibly related:** An event that follows a reasonable temporal sequence from the initiation of study procedures, but that could readily have been produced by a number of other factors.

**Related:** The AE is clearly related to the study procedures.

**MANAGEMENT OF RISKS TO SUBJECTS**

Expected AEs

Expected AEs associated with the placental transfusion include:

MATERNAL- Postpartum haemorrhage (PPH)

NEONATAL- Polycythemia, Hyperbilirubinaemia and need for phototherapy

AE Management

**All adverse events will be reported to ERC within 24 hours.**

Maternal AE: To prevent PPH, AMTSL with administration of IM oxytocin will be included in all deliveries, labour ward is also equipped with other Uterotonics and a theatre in case PPH still occurs.

Midwives in labour ward are well trained to manage PPH.

Neonatal AE: The infant will be under continuous monitoring by the nurse and paediatrician in the respective wards. Kenyatta National Hospital is equipped to provide phototherapy for infants that require the intervention as well as if exchange transfusion is required.

**DATA ANALYSIS PLANS**

The statistician will not be blinded and data monitoring will be continuous, he will alert the principal investigator of any overt differences in the two groups. The study will be halted if there is evidence of significant statistical advantage in whichever group, to avoid denying the corresponding group an advantageous intervention.

In case of SAE, a report will be forwarded to ERC and study halted immediately pending clearance.

**PLAN FOR DATA MANAGEMENT**

Compliance of regulatory documents and study data accuracy and completeness will be maintained through an internal study team quality assurance process.

Confidentiality throughout the trial is maintained by assigning a code to each participant, for purposes of identification. The key, linking the patient to the identifying code will be stored separately from the research data, in a password-protected database. This will only be accessible to the principal investigator.

## APPENDIX V: LINK LOG

**Study Title:** **THE EFFECT OF UMBILICAL CORD MILKING VERSUS DELAYED CORD CLAMPING ON SELECTED HAEMATOLOGICAL AND CLINICAL PARAMETERS IN PRETERM NEONATES AT KENYATTA NATIONAL HOSPITAL.**

| DATE | NAME | MOBILE NUMBER/  NEXT OF KIN NUMBER | ENROLLMENT IDENTIFICATION NUMBER |
| --- | --- | --- | --- |
| **1** |  |  |  |
| **2** |  |  |  |
| **3** |  |  |  |
| **4** |  |  |  |
| **5** |  |  |  |
| **6** |  |  |  |
| **7** |  |  |  |
| **8** |  |  |  |
| **9** |  |  |  |
| **10** |  |  |  |
