# Supplementary material for: Effect of umbilical cord milking versus delayed cord clamping on preterm neonates in Kenya: A randomized controlled trial
Source: PLoS One. 2021 Jan 26;16(1):e0246109. doi: 10.1371/journal.pone.0246109 (PMC7837492; doi:10.1371/journal.pone.0246109)
Supplement: S1 Data — (DOCX) [file pone.0246109.s003.docx]

**Availability of data:** https://doi.org/10.5061/dryad.8pk0p2nm2
